# Basement membrane proteins in extracellular matrix characterize NF1 neurofibroma development and response to MEK inhibitor

**DOI:** 10.1172/JCI168227

**Published:** 2023-06-15

**Authors:** Chunhui Jiang, Ashwani Kumar, Ze Yu, Tracey Shipman, Yong Wang, Renee M. McKay, Chao Xing, Lu Q. Le

**Affiliations:** 1 Department of Dermatology,; 2 Eugene McDermott Center for Human Growth and Development,; 3 Lyda Hill Department of Bioinformatics,; 4 Simmons Comprehensive Cancer Center,; 5 UTSW Comprehensive Neurofibromatosis Clinic,; 6 Hamon Center for Regenerative Science and Medicine, and; 7 O’Donnell Brain Institute, University of Texas Southwestern Medical Center at Dallas, Dallas, Texas, USA.

**Keywords:** Oncology, Extracellular matrix, Macrophages, Tumor suppressors

## Abstract

Neurofibromatosis type 1 (NF1) is one of the most common tumor-predisposing genetic disorders. Neurofibromas are NF1-associated benign tumors. A hallmark feature of neurofibromas is an abundant collagen-rich extracellular matrix (ECM) that constitutes more than 50% of the tumor dry weight. However, little is known about the mechanism underlying ECM deposition during neurofibroma development and treatment response. We performed a systematic investigation of ECM enrichment during plexiform neurofibroma (pNF) development and identified basement membrane (BM) proteins, rather than major collagen isoforms, as the most upregulated ECM component. Following MEK inhibitor treatment, the ECM profile displayed an overall downregulation signature, suggesting ECM reduction as a therapeutic benefit of MEK inhibition. Through these proteomic studies, TGF-β1 signaling was identified as playing a role in ECM dynamics. Indeed, TGF-β1 overexpression promoted pNF progression in vivo. Furthermore, by integrating single-cell RNA sequencing, we found that immune cells including macrophages and T cells produce TGF-β1 to induce Schwann cells to produce and deposit BM proteins for ECM remodeling. Following *Nf1* loss, neoplastic Schwann cells further increased BM protein deposition in response to TGF-β1. Our data delineate the regulation governing ECM dynamics in pNF and suggest that BM proteins could serve as biomarkers for disease diagnosis and treatment response.

## Graphical abstract

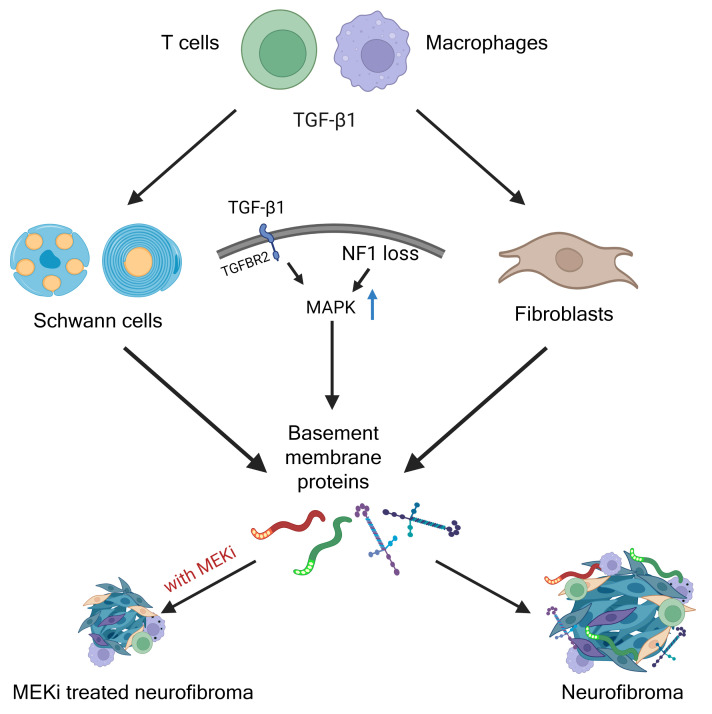

## Introduction

Neurofibromatosis type 1 (NF1) is a genetic disease of the nervous system that affects about 1 in 3,000 individuals worldwide. It is caused by mutation in the *NF1* gene, which encodes the protein neurofibromin, a negative regulator of the RAS/MAPK signaling pathway. NF1 patients display a wide spectrum of clinical manifestations, including café au lait macules, axillary or inguinal freckling, optic gliomas, and neurofibromas. Neurofibromas are benign nerve sheath tumors that develop in the peripheral nervous system. There are 2 types of neurofibromas: (a) cutaneous neurofibromas (cNFs) grow along nerve twigs in the skin, while (b) plexiform neurofibromas (pNFs) grow along nerve plexus deep in the body. Both stem from *NF1* loss in Schwann cell–lineage cells. Treatment for neurofibroma is limited, and surgical resection remains the mainstay intervention. However, selumetinib, a MEK inhibitor (MEKi), was approved by the FDA in 2020 for inoperable pNF (1). The mechanism by which MEK inhibition exerts its therapeutic effects has been primarily attributed to inhibition of cell proliferation (2), but other effects on tumor cell physiology may also be involved. Further exploration of its mechanism of action could potentially advance neurofibroma treatment.

Neurofibromas are heterogeneous tumors composed of neoplastic Schwann cells, fibroblasts, immune cells, neurons, and endothelial cells, as well as a collagen-rich ECM network. Evidence from genetically engineered mouse models (GEMMs) demonstrated that loss of *Nf1* in a spatiotemporal manner during Schwann cell development gives rise to neurofibromas (3–12). Schwann cells are derived from a subset of neural crest stem cells (NCSCs) that migrate from the neural tubes to the periphery. Once in contact with neuronal axons, NCSCs differentiate into Schwann cell precursors and then into mature myelinating and non-myelinating Schwann cells. Further characterization of these cell populations with novel markers will provide critical insights to unravel neurofibroma tumorigenesis.

The role of the tumor microenvironment is also key to fully understanding neurofibroma development and treatment response (13, 14). Compared with the cellular components within the tumor microenvironment, ECM is less well characterized regarding its roles in neurofibroma development and treatment response. Fibroblasts have been regarded as the principal source of ECM deposition not only under normal physiological conditions, such as during wound healing, but also in pathological contexts, such as organ fibrosis and cancer. Under both circumstances, fibroblasts can be activated to become myofibroblasts with enhanced ECM production (15). However, neurofibroma-associated fibroblasts lack expression of classic fibrogenic markers, such as smooth muscle actin (16), suggesting that the neurofibroma ECM may have a unique signature. Characterization of ECM profiles in neurofibroma may be informative in understanding the contribution of tumor microenvironment in neurofibroma pathogenesis as well as in therapeutic intervention.

As abundant ECM accumulation is observed in neurofibroma, it is important to elucidate whether and how neoplastic Schwann cells remodel the ECM network during tumor development and treatment response. During peripheral nerve development, Schwann cells actively deposit ECM, which plays essential roles in regulating neuron and Schwann cell physiology (17, 18). Electron microscopy has revealed that Schwann cells are surrounded by basement membranes (BMs), thin but dense sheets of specialized ECM structure composed of independent type IV collagen and laminin polymeric networks. Studies from decades ago showed an extensive BM network around Schwann cells and blood vessels in neurofibroma (19). However, the mechanisms regulating ECM deposition by neoplastic Schwann cells in neurofibroma remain to be explored.

Here, we performed ECM proteomic studies and found that BM proteins increase during pNF development and decrease with MEKi treatment. Integrating these data with data from single-cell RNA sequencing (scRNA-Seq), we found that Schwann cells contribute significantly to ECM dynamics by depositing BM proteins. We also found that immune cells including macrophages and T cells produce TGF-β1 to promote ECM remodeling. Mechanistically, we have identified a regulatory network whereby *NF1* loss and TGF-β1 upregulation collectively lead to BM protein deposition by Schwann cells.

## Results

### Defining the ECM profile during pNF development.

**Figure 1:**
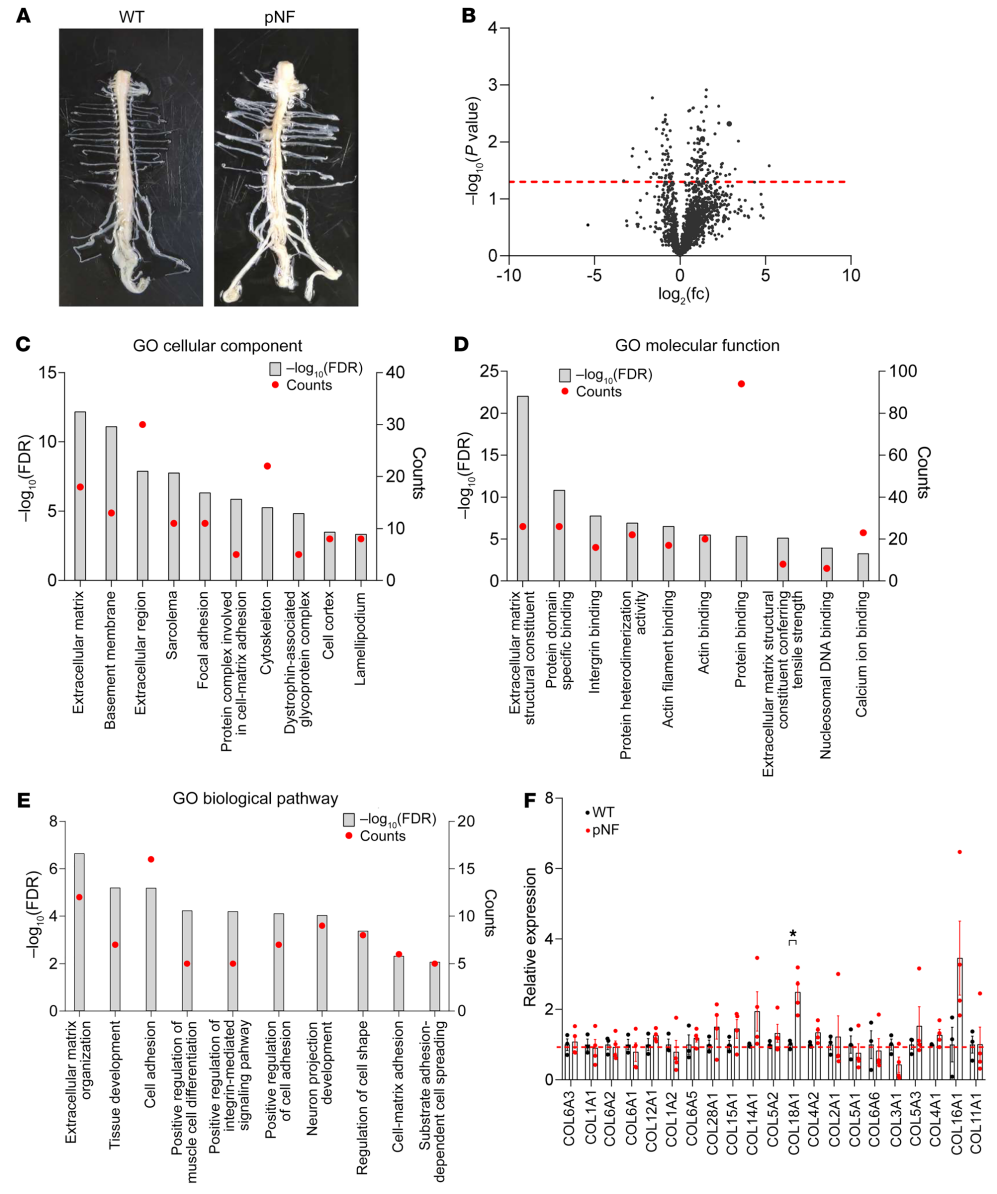
Plexiform neurofibroma development is characterized by ECM enrichment. (**A**) Representative images showing spinal cords extracted from wild-type (WT) mice (*n* = 3) and *H7;Nf1mut* mice, which develop plexiform neurofibroma (pNF) (*n* = 4). The pNF spinal cord shows enlarged dorsal root ganglia (DRGs), indicating pNF formation. (**B**) Volcano plot of the mass spectrometry data set showing *P* values against fold changes (fc). The red line indicates *P* value equal to 0.05, and targets above the red line are significantly changed. (**C**–**E**) Gene Ontology (GO) analysis showing the top 10 significantly upregulated categories in cellular component (**C**), molecular function (**D**), and biological pathway (**E**) in pNF compared with WT. The left *y* axis indicates the level of significance of each category. FDR, false discovery rate. The right *y* axis indicates the number of targets included in each category. (**F**) Mass spectrometry data analysis for the indicated collagen isoforms based on the abundance ratios. Data are presented as means ± SEM. Comparisons among groups were performed by Student’s *t* test. *P < 0.05.

As ECM accumulation is a hallmark feature of neurofibroma, we set out to define the components of the ECM and to understand their role in neurofibroma biology. To do this, we first isolated spinal cords and extracted dorsal root ganglia (DRGs) (Figure 1A), where pNFs develop in GEMMs, from *Hoxb7-Cre Nf1^fl/fl^* and *Hoxb7-Cre Nf1^fl/–^* mice (*H7;Nf1mut*) (*n* = 4); by 12 months of age, pNFs have typically already formed in these mutant mice as reported in previous studies (10). DRGs from age-matched wild-type (WT) mice (*n* = 3) were used as control. Protein was extracted from DRGs, and ECM protein was enriched using a CNMCS Compartmental Protein Extraction Kit and then subjected to mass spectrometry. Mass spectrometry results showed that 1,094 of 1,588 targets were increased and 91 targets were significantly upregulated in pNF samples (Figure 1B). We then input these 91 targets into the Database for Annotation, Visualization and Integrated Discovery (DAVID; https://david.ncifcrf.gov/tools.jsp) for Gene Ontology (GO) analysis. The GO cellular component analysis showed that multiple ECM-relevant categories were highlighted, including ECM, BM, extracellular region, protein complex involved in cell-matrix adhesion, and dystrophin-associated glycoprotein complex (Figure 1C). Conversely, 494 targets were decreased and 48 targets were significantly reduced in pNF samples. The GO cellular component analysis of these 48 targets showed that myelin sheath and neurofilament were decreased in pNF samples (Supplemental Figure 1A; supplemental material available online with this article; https://doi.org/10.1172/JCI168227DS1), suggesting the underrepresentation of normal myelinating Schwann cells and neurons in pNF DRGs. Additionally, the GO molecular function analysis of the 91 upregulated targets in pNF samples highlighted multiple ECM-related categories, such as ECM structural constituent, integrin binding, and ECM structural constituent conferring tensile strength (Figure 1D). The GO biological pathway analysis showed upregulation of ECM-related pathways in the pNF group, including ECM organization, cell adhesion, positive regulation of integrin-mediated signaling pathways, positive regulation of cell adhesion, cell-matrix adhesion, and substrate adhesion-dependent cell spreading (Figure 1E). These results are consistent with pNF biological features and, collectively, validate our analytical pipeline. As collagen enrichment is one of the key pathological features in pNF diagnosis, we analyzed the collagen profile in our data sets. After normalization to total protein abundance, the collagen isoforms exhibited varied expression levels in pNF compared with WT, with Col18a1 as the only significantly upregulated isoform (Figure 1F). We also analyzed ECM regulators based on the mouse matrisome database, MatrisomeDB (20), and found that multiple ECM regulators were significantly upregulated in pNF, including ITIH1, ITIH5, SERPINH1, F13A1, and TGM2 (Supplemental Figure 1B). Altogether, these results demonstrate the enrichment of ECM in pNF.

### MEK inhibitor decreases ECM deposition in pNF.

**Figure 2:**
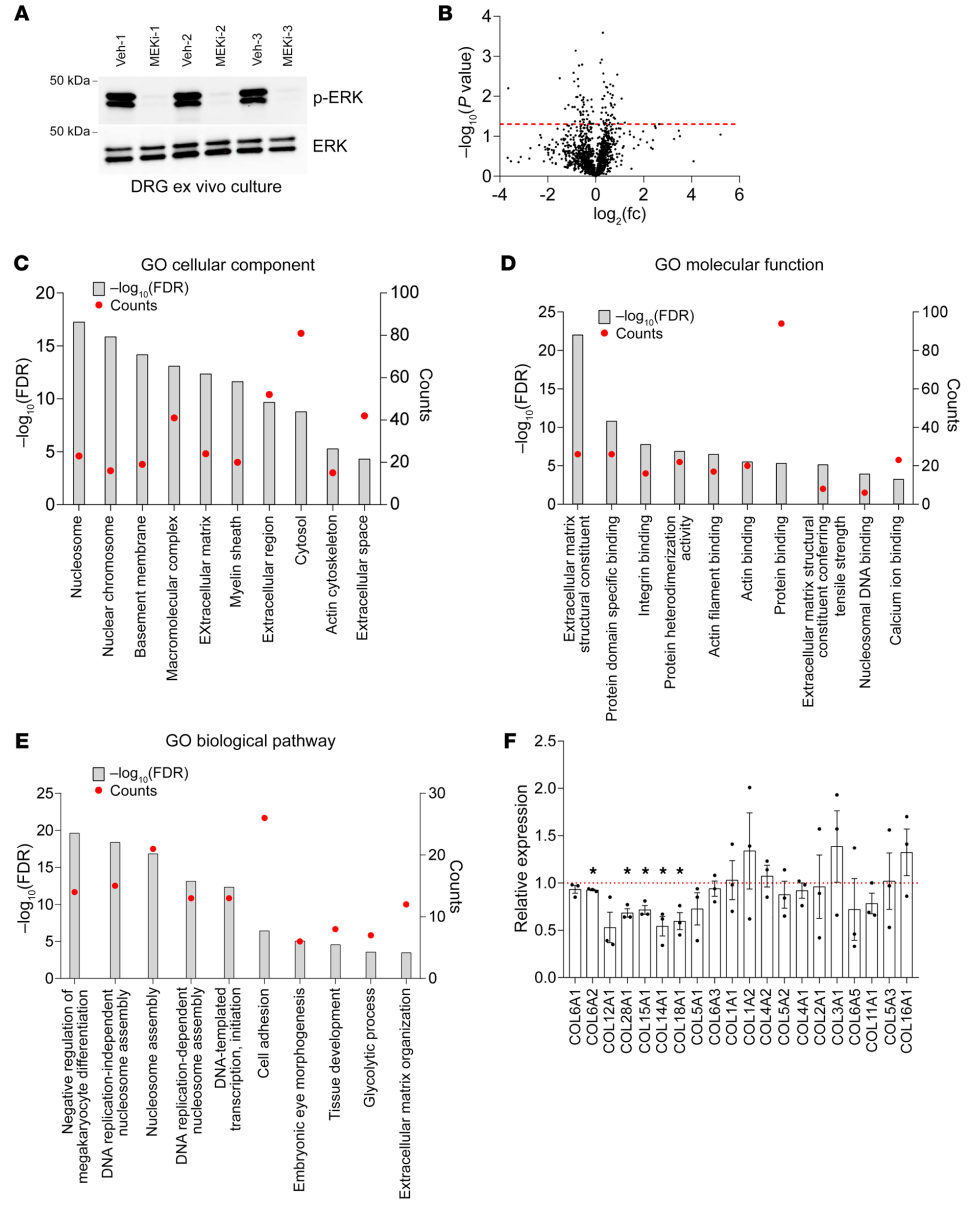
MEK inhibitor treatment of pNF decreases ECM deposition. (**A**) Western blots of DRGs extracted from *H7;Nf1mut* mice, cultured ex vivo, and treated with vehicle (Veh) (*n* = 3) or MEK inhibitor (MEKi) (*n* = 3) for 3 days. (**B**) Volcano plot of the mass spectrometry data set showing *P* values against fold changes (fc). The red line indicates *P* value equal to 0.05, and targets above the red line are significantly changed. (**C**–**E**) GO analysis showing the top 10 significantly downregulated categories in cellular component (**C**), molecular function (**D**), and biological pathway (**E**) in the MEKi-treated groups compared with vehicle-treated. The left *y* axis indicates the level of significance of each category. FDR, false discovery rate. The right *y* axis indicates the number of targets included in each category. (**F**) Mass spectrometry data analysis of the indicated collagen isoforms based on the abundance ratios. Data are presented as the ratios of MEKi compared with vehicles. Data are shown as means ± SEM. Comparisons among groups were performed by Student’s *t* test. *P < 0.05.

As MEK inhibition has been approved by the FDA as a treatment strategy for inoperable pNF, we next examined how the ECM in pNF changes in response to MEK inhibitor (MEKi) treatment. DRGs were isolated from *H7;Nf1mut* mice at 6–8 months of age and split into 2 groups for ex vivo culture: one group was treated with the MEKi PD0325901 and the other with vehicle (*n* = 3). Protein was extracted from DRGs after 3 days, and treatment efficacy was verified by Western blot for phospho-ERK levels, which were greatly reduced with MEKi treatment (Figure 2A). ECM protein was enriched with the CNMCS Compartmental Protein Extraction Kit, followed by mass spectrometry. The same analysis principles as in Figure 1 were applied, and 1,291 targets were selected with a threshold of 10 peptide-spectrum matches. The average fold changes in the 3 pairwise comparisons were calculated, and 729 of 1,291 targets were decreased in the MEKi-treated group compared with vehicle (Figure 2B). One hundred ninety targets were consistently decreased in all 3 MEKi-treated samples. We then input these 190 targets into DAVID for GO analysis. The GO cellular component analysis emphasized BM, ECM, extracellular region, and extracellular space (Figure 2C). Consistently, the GO molecular function analysis highlighted ECM structural constituent, integrin binding, and ECM structural constituent conferring tensile strength (Figure 2D). In addition, the GO biological pathway analysis highlighted cell adhesion and ECM organization (Figure 2E). Thus, the GO analysis confirmed reduced ECM deposition following MEKi treatment. Furthermore, we also examined the expression profile of multiple collagens after MEKi treatment and found that several collagen isoforms were significantly decreased by MEKi treatment, including COL6A2, COL28A1, COL15A1, COL14A1, and COL18A1 (Figure 2F). On the contrary, neuron- and myelination-related targets were found to be increased after MEKi treatment (Supplemental Figure 2A), consistent with the reduction of neoplastic Schwann cells by MEKi treatment and corresponding upregulation of neuron and myelination targets, further validating our data analysis pipeline. We also analyzed ECM regulators and found that ITIH2 was significantly decreased by MEKi treatment (Supplemental Figure 2B). In summary, we found that MEKi treatment reduced ECM deposition in pNF, which likely contributes to its therapeutic efficacy.

### ECM dynamics reveal TGF-β1 regulation of ECM deposition in pNF.

**Figure 3:**
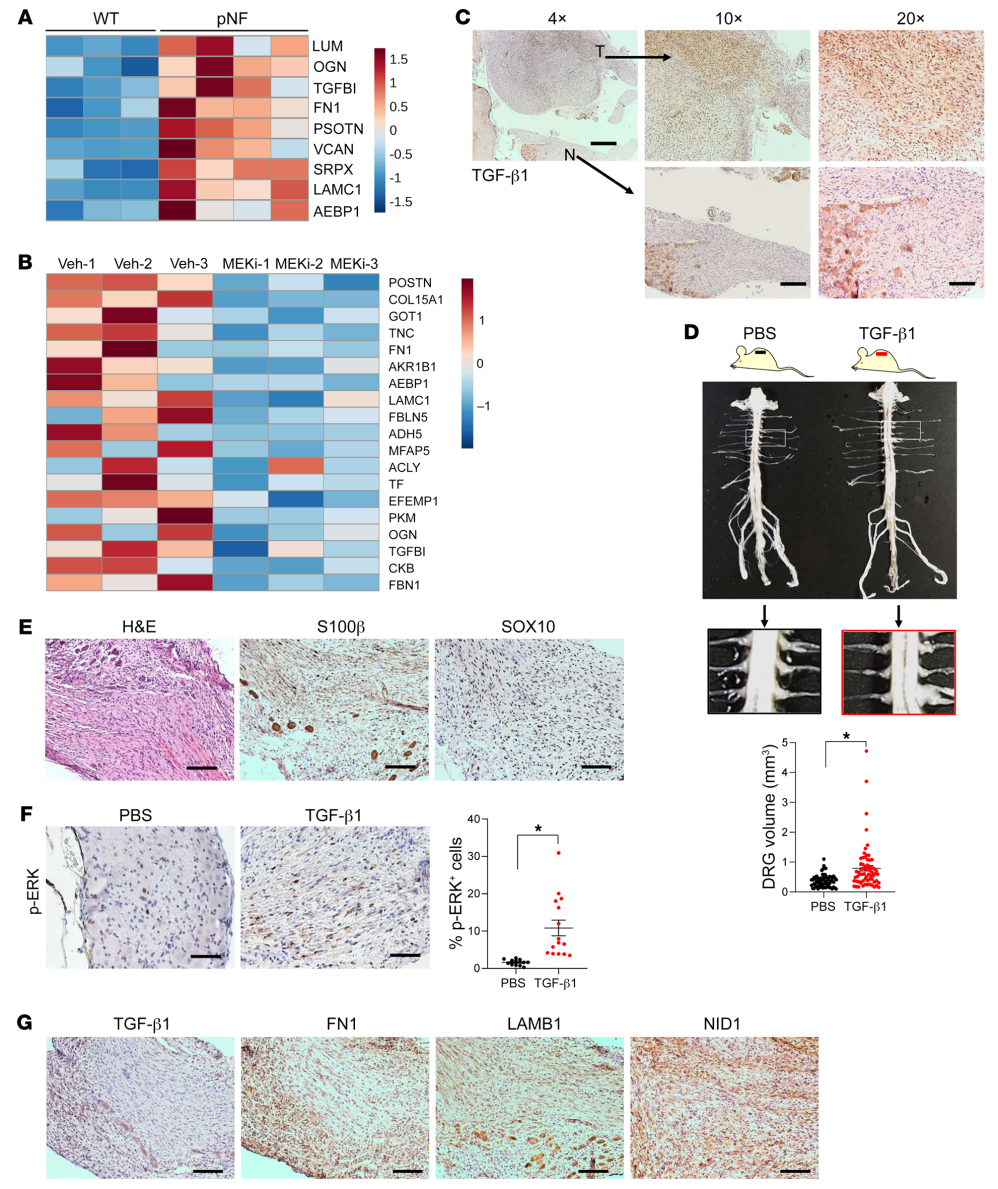
ECM dynamics reveal TGF-β1 regulation of ECM deposition in pNF. (**A**) Heatmap showing enrichment of TGF-β–related targets in pNF compared with WT by mass spectrometry quantification. Expression levels were *z* score–normalized by row. These targets are predicted to be involved in the biological pathway of TGF-β regulation of ECM based on the BioPlanet 2019 database. (**B**) Heatmap visualization of mass spectrometry quantification showing the TGF-β–related targets decreased in MEKi-treated compared with vehicle-treated DRGs from *H7;Nf1mut* mice. Expression levels were *z* score–normalized by row. These targets are predicted to be involved in the biological pathway of TGF-β regulation of ECM based on the BioPlanet 2019 database. (**C**) Representative immunohistochemistry images showing expression of TGF-β1 in pNF (T) and normal (N) DRGs on the same tissue section. Scale bars: 500 μm in ×4 image; 200 μm in ×10 images; 100 μm in ×20 images. *n* = 3 pairs of mice. (**D**) Representative images showing spinal cords extracted from *H7;Nf1mut* mice implanted with PBS- or TGF-β1–releasing capsules. The spinal cord from TGF-β1–treated mice shows enlarged DRGs, indicating pNF formation. DRG volumes were measured and were significantly larger in mice harboring TGF-β1–releasing capsules. *n* = 3 pairs of mice with 19–24 DRGs quantified for each mouse. (**E**) Representative images showing H&E staining and S100β and SOX10 immunohistochemistry of DRG sections from TGF-β1–treated mice. *n* = 3 pairs of mice. (**F**) Representative images showing immunohistochemistry for phospho-ERK (p-ERK) expression in DRGs from PBS- and TGF-β1–treated groups. Ratios of p-ERK^+^ cells were quantified. *n* = 3 pairs of mice with 4–6 images quantified for each mouse. (**G**) Representative images showing immunohistochemistry of TGF-β1, FN1, LAMB1, and NID1 in DRGs from the TGF-β1 group. *n* = 3 pairs of mice. Scale bars: 100 μm. Data are shown as means ± SEM. Comparisons among groups were performed by nested *t* test. *P < 0.05.

Our 2 mass spectrometry data sets demonstrate a correlation between ECM deposition and pNF growth and treatment response: ECM deposition is increased in pNF compared with WT tissue and is decreased upon MEKi treatment. To investigate the mechanism underlying this correlation, we input the 91 targets increased in pNF compared with WT and the 190 targets decreased with MEKi treatment into the Enrichr website (https://maayanlab.cloud/Enrichr/), respectively, and searched for enrichment of biological pathways. With the BioPlanet 2019 database, the biological pathway of “TGF-beta regulation of extracellular matrix” was identified for both data sets. In the 91 targets increased in pNF, the target profile reflecting “TGF-beta regulation of extracellular matrix” includes SRPX, POSTN, VCAN, LUM, OGN, FN1, LAMC1, AEBP1, and TGFBI (Figure 3A). In the 190 targets decreased following MEKi treatment, the target profile reflecting the same pathway included POSTN, COL15A1, GOT1, TNC, FN1, AKR1B1, AEBP1, LAMC1, FBLN5, ADH5, MFAP5, ACLY, TF, EFEMP1, PKM, OGN, TGFBI, CKB, and FBN1 (Figure 3B). We next verified the expression of TGF-β1 with immunohistochemistry in pNF samples. The expression level of TGF-β1 in the pNF DRGs was more abundant in comparison with the adjacent normal DRGs (Figure 3C). We also confirmed the upregulation of TGF-β1 in the E13.5 DRG neurosphere cell transplantation model, showing that the expression level of TGF-β1 was higher in the pNF region compared with the normal sciatic nerve region (Supplemental Figure 3). These immunohistochemistry data are consistent with previous publications regarding hyperactivation of TGF-β1 in neurofibroma both in vitro and in vivo (21–24).

We next wanted to determine whether TGF-β1 hyperactivation actually contributes to pNF pathogenesis in our mouse models. Previous studies demonstrated that TGF-β1 induces NF1 patient–derived fibroblasts to form neurofibroma-like phenotypes in vitro, including excessive collagen deposition (21). However, such evidence from in vivo experiments is lacking. Thus, we implanted TGF-β1–releasing capsules into *H7;Nf1mut* mice at 4–6 months of age, when they have just begun to develop neurofibroma. After 1 month, we isolated spinal cords and characterized pNF progression. We found that the mice implanted with TGF-β1 capsules had significantly larger DRGs than the control *H7;Nf1mut* mice implanted with PBS capsules (Figure 3D). Furthermore, the acceleration of pNF progression by TGF-β1 treatment was confirmed by H&E staining and immunohistochemistry, which revealed hypercellularity and disorganized structure, as well as expression of Schwann cell markers S100β and SOX10, respectively (Figure 3E). Moreover, the expression level of phospho-ERK was significantly increased by TGF-β1 treatment (Figure 3F), suggesting that TGF-β1 may be an upstream regulator of the RAS/MAPK pathway. In addition, TGF-β1 and several ECM components, including FN1, LAMB1, and NID1, were abundantly expressed in the DRGs with TGF-β1 capsule implantation (Figure 3G). These data suggest that TGF-β1 upregulation promotes pNF progression in vivo.

### Schwann cells express BM proteins that contribute to ECM deposition in pNF.

**Figure 4:**
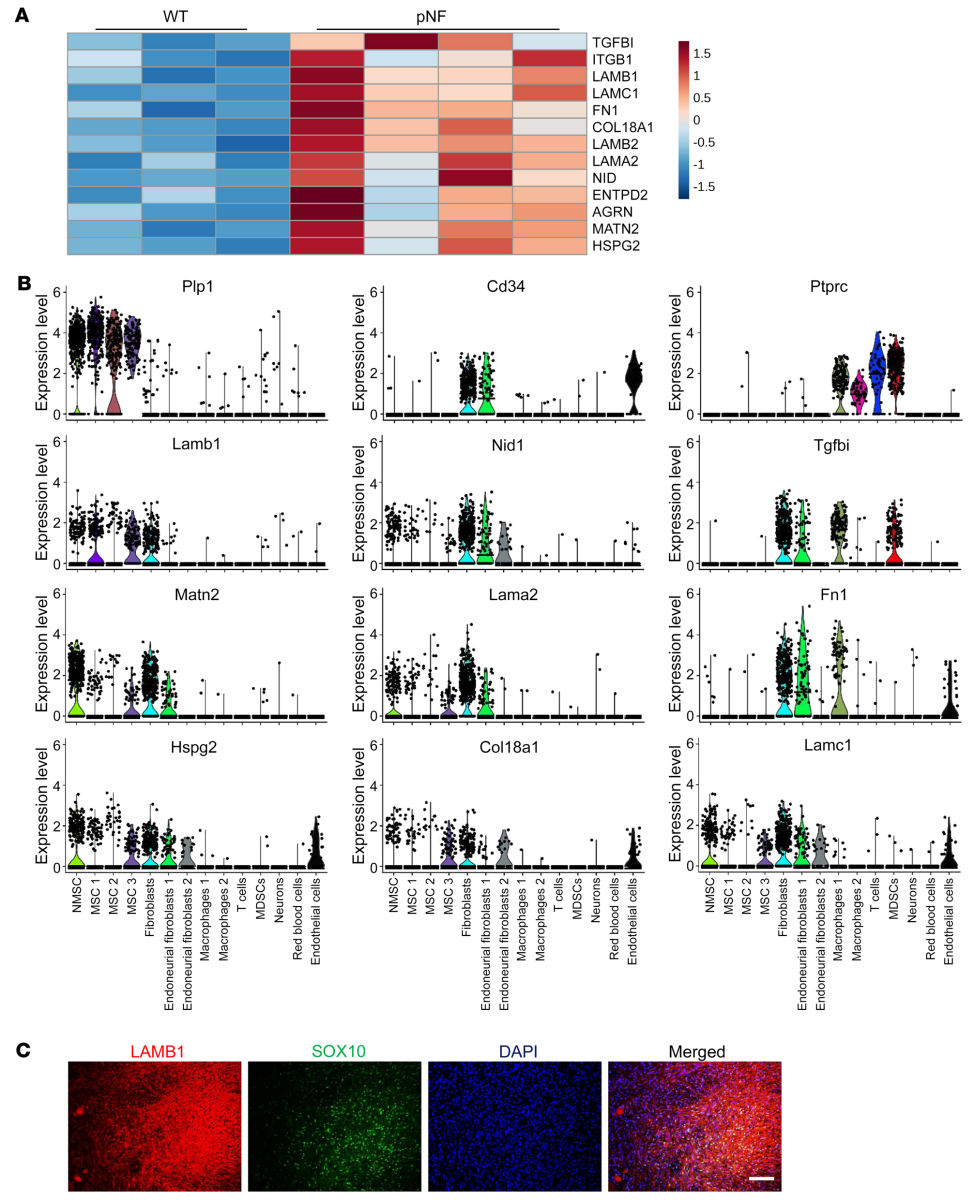
Schwann cells express BM proteins that contribute to ECM deposition in pNF. (**A**) Heatmap of mass spectrometry quantification showing BM targets enriched in pNF compared with WT DRGs. The expression levels were *z* score–normalized by row. These BM targets were identified in the GO cellular component analysis shown in Figure 1C. (**B**) Violin plots showing the expression levels of indicated targets. NMSC, non-myelinating Schwann cells; MSC, myelinating Schwann cells; MDSC, myeloid-derived suppressor cells. (**C**) Representative images showing the coimmunofluorescence of LAMB1 and SOX10 in mouse pNF tissue. Scale bar: 100 μm.

To understand the underlying mechanism by which TGF-β1 hyperactivation contributes to pNF pathogenesis, we investigated whether and how different types of cells in pNF respond to hyperactivation of TGF-β1. We revisited our mass spectrometry results with the GO cellular component analysis (Figure 1C and Figure 2C) and found that those targets reflecting ECM dynamics during pNF and treatment response highlight a profile of a specific ECM structure: BM. Several BM proteins were observed to be enriched in pNF, including LAMB1, LAMC1, LAMB2, LAMA2, NID1, HSPG2, AGRN, TGFBI, ITGB1, ENTPD2, MATN2, FN1, and COL18A1 (Figure 4A).

To characterize the cellular sources of these BM proteins, as well as gain insights into the regulation of ECM deposition by TGF-β1 in pNF, we performed scRNA-Seq experiments with mouse pNF tissues. Cells were isolated from *H7;Nf1mut* mouse DRGs, and then subjected to scRNA-Seq using 10x Genomics technology. By comparison of the marker lists recently characterized in *Dhh-Cre Nf1^fl/fl^* pNF mouse models (25), 14 major cell type clusters were identified, including Schwann cells, fibroblasts, immune cells, and neurons (Supplemental Figure 4A). We first analyzed the cellular expression of BM components. It was not surprising to observe that *Cd34^+^* fibroblasts produce multiple BM proteins in pNF. However, *Plp1^+^* Schwann cells also contribute to deposition of a number of BM proteins, including *Lamb1*, *Nid1*, *Matn2*, *Hspg2*, *Lamc1*, *Lama2*, and *Col18a1*, but not *Fn1* and *Tgfbi*, both of which were predominantly expressed by *Cd34*^+^ fibroblasts and *Ptprc*^+^ immune cells (Figure 4B and Supplemental Figure 4B). We confirmed our scRNA-Seq data with coimmunofluorescence in mouse pNF tissue and found that LAMB1 was indeed colocalized with SOX10^+^ Schwann cells (Figure 4C) while FN1 was less colocalized (Supplemental Figure 4C). Moreover, we also compared BM protein expression in Schwann cells and fibroblasts. Although fibroblasts have an overall higher capacity for BM protein deposition, Schwann cells deposit specific BM proteins at comparable levels, such as *Lamb1*, *Matn2*, *Col18a1*, and *Hspg2* (Supplemental Figure 4B). We also verified BM protein expression in primary Schwann cells and fibroblasts isolated from mouse pNF DRGs. Cells were collected at early passages (passage 0–2) to minimize the effects of cell culture. Both types of cells express LAMB1 and NID1, with higher expression in fibroblasts, consistent with our scRNA-Seq analysis (Supplemental Figure 4D). Additionally, we examined the expression of ECM regulators identified in our mass spectrometry data sets and found that Schwann cells express multiple peptidases, proteases, protease inhibitors, and other regulators (Supplemental Figure 5), suggesting an active role for Schwann cells in ECM remodeling. Notably, several ECM regulators, such as *Itih5*, were predominantly expressed by Schwann cells, which warrants further investigation. In summary, these data demonstrate that Schwann cells contribute to ECM remodeling in pNF.

### Macrophages and T cells secrete TGF-β1 in pNF.

**Figure 5:**
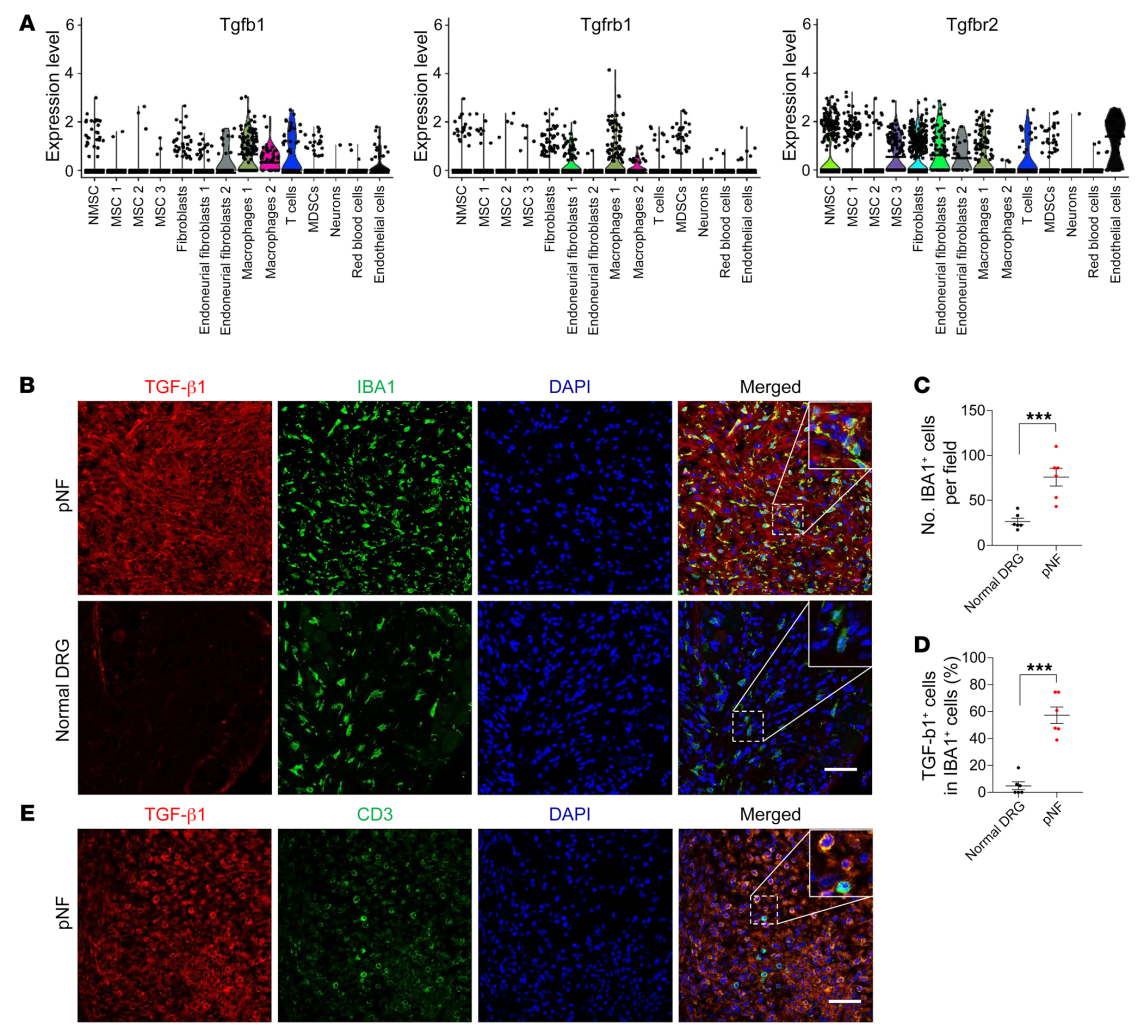
Macrophages and T cells secrete TGF-β1 in pNF. (**A**) Violin plots showing the expression levels of TGF-β1 ligand and receptors in different cell populations. NMSC, non-myelinating Schwann cells; MSC, myelinating Schwann cells; MDSC, myeloid-derived suppressor cells. (**B**) Immunofluorescence images showing coimmunofluorescence of TGF-β1 and IBA1 in mouse pNF tissue and adjacent normal DRG tissue. Insets show higher magnification. Scale bar: 50 μm. (**C**) Graphical analysis of **B** showing the number of IBA1^+^ cells per field in mouse pNF tissue and adjacent normal DRG tissue. *n* = 6. (**D**) Graphical analysis of **B** showing the ratios of TGF-β1^+^ cells in IBA1^+^ cells in mouse pNF tissue and adjacent normal DRG tissue. *n* = 6. (**E**) Immunofluorescence images showing coimmunofluorescence of TGF-β1 and CD3 in mouse pNF tissue and adjacent normal DRG tissue. Insets show higher magnification. Scale bar: 50 μm. Data are shown as means ± SEM. Comparisons among groups were performed by Student’s *t* test. ***P < 0.001.

Using the scRNA-Seq data set, we also analyzed TGF-β1 ligand and receptor expression. TGF-β1 initiates signaling in the pathway by binding to the TGF-β1 receptor complex composed of type I (TGFBR1) and type II (TGFBR2) subunits. We found that *Tgfb1* is predominantly expressed by immune cells, including macrophages and T cells (Figure 5A). To verify, we performed coimmunofluorescence experiments and found that TGF-β1 was indeed colocalized with macrophage marker IBA1 in pNF tissues (Figure 5B). We also found that the number of macrophages was much higher in pNF compared with normal DRG tissue (Figure 5C), consistent with a previous report showing that the number of macrophages correlates with neurofibroma progressive disease severity, from normal phenotype to neurofibroma to malignant peripheral nerve sheath tumor (14). Interestingly, a large fraction of IBA1^+^ macrophages in pNF express a much higher level of TGF-β1 than IBA1^+^ macrophages in normal DRG tissues (Figure 5, B and D). In addition, we found that TGF-β1 was colocalized with T cell marker CD3 in pNF tissues (Figure 5E). CD3^+^ T cells were barely detectable in normal DRG tissues but were induced in pNF tissues (Figure 5E), consistent with a previous publication showing that T cells are necessary for neurofibroma formation (26). In addition, Schwann cells express the TGF-β1 receptors *Tgfbr1* and *Tgfbr2* (Figure 5A), suggesting that Schwann cells may respond to TGF-β1 by depositing ECM components in pNF. Altogether, these data collectively suggest a previously unappreciated role for macrophages and T cells in pNF tumorigenesis.

### Expression levels of BM proteins change with pNF growth and treatment.

**Figure 6:**
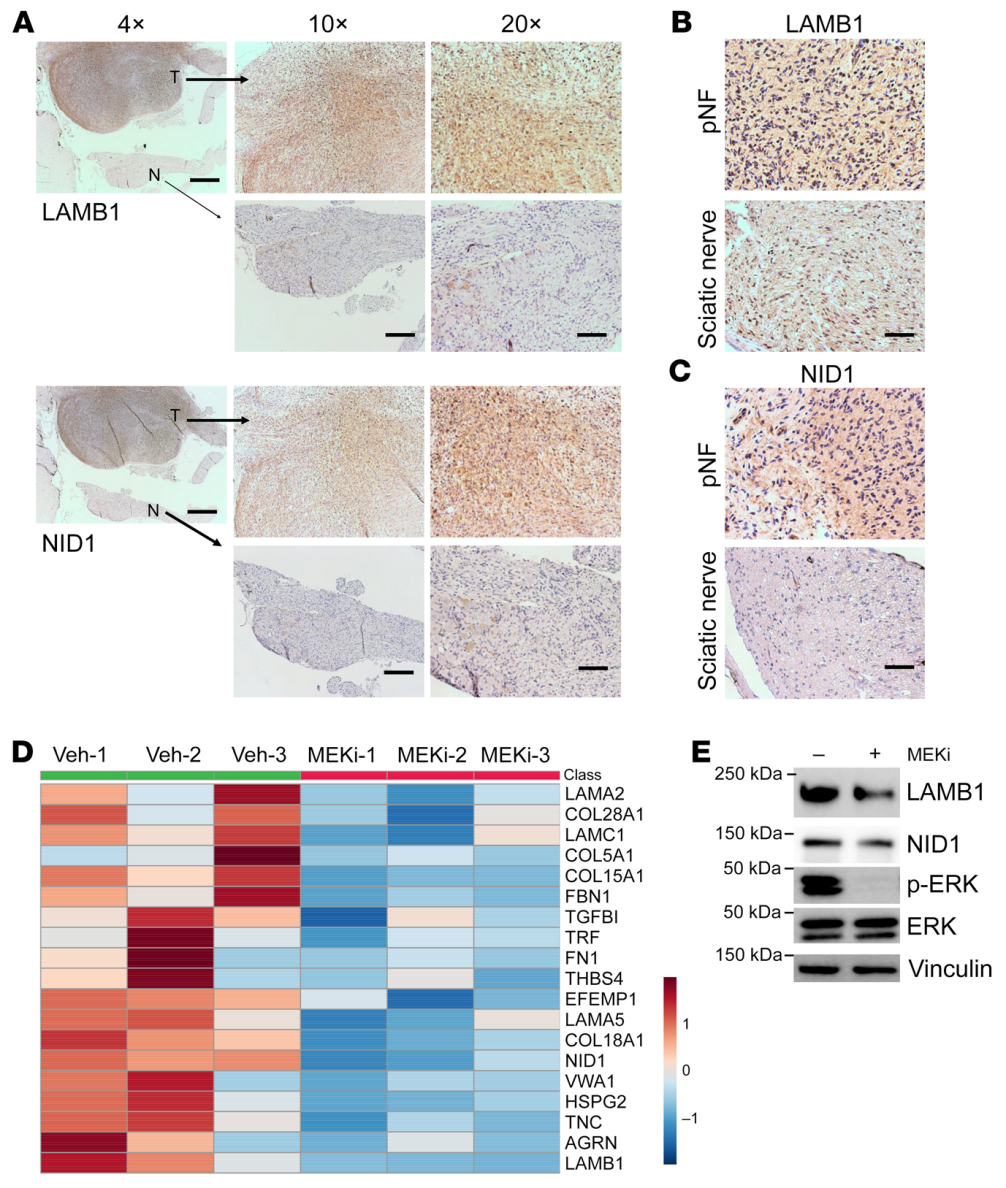
Expression levels of BM proteins change with pNF growth and treatment. (**A**) Representative immunohistochemistry images showing LAMB1 and NID1 expression in pNF (T) and normal (N) DRGs on the same tissue section. Scale bars: 500 μm in ×4 image; 200 μm in ×10 images; 100 μm in ×20 images. (**B** and **C**) Representative immunohistochemistry images showing LAMB1 (**B**) and NID1 (**C**) expression in human pNF tissue and normal sciatic nerve tissue. Scale bars: 100 μm. (**D**) Heatmap showing the BM targets identified in mass spectrometry quantification of vehicle- and MEKi-treated DRGs. The expression levels were *z* score–normalized by row. These BM targets were identified in the GO cellular component analysis shown in Figure 2C. (**E**) Representative Western blots showing expression levels of the indicated proteins in DRGs treated with vehicle or MEKi ex vivo. *n* = 3.

To further characterize BM proteins in pNF, we examined their abundance levels based on our mass spectrometry results. The 91 targets identified to be significantly enriched in pNF were ranked based on their abundance ratios, which were calculated as the percentages of target protein abundances in the total protein abundances. Interestingly, the top 7 upregulated ECM proteins in pNF were all BM proteins: LAMA2, HSPG2, LAMC1, LAMB2, TGFBI, NID1, and LAMB1 (Table 1). Given that these BM proteins can be deposited by Schwann cells, these results are consistent with the increase of Schwann cell number in pNF. We verified the enrichment of LAMB1 and NID1 with immunohistochemistry, which showed a dramatic accumulation in pNF DRGs compared with adjacent normal DRGs (Figure 6A). We also examined the expression of LAMB1 and NID1 in human pNF tumor samples and found that both were abundantly deposited in human pNF samples compared with normal sciatic nerve (Figure 6, B and C). These results collectively verified the enrichment of BM proteins in pNF. Notably, both LAMB1 and NID1 were also enriched in mouse and human cNF compared with normal skin tissues (Supplemental Figure 6, A and B).

On the other hand, the BM category was also highlighted in the GO cellular component analysis of the 190 significantly decreased targets following MEKi treatment (Figure 2C). There were 19 BM proteins that were reduced by MEKi treatment: LAMB1, LAMA2, LAMA5, LAMC1, NID1, TGFBI, HSPG2, FN1, AGRN, COL18A1, COL5A1, COL15A1, COL28A1, EFEMP1, FBN1, TNC, TRF, THBS4, and VWA1 (Figure 6D). By Western blot analysis, we confirmed the reduced expression of LAMB1 and NID1 following MEKi treatment in the ex vivo DRG cultures from *H7;Nf1mut* mice (Figure 6E). Combined with the upregulation of BM proteins in pNF development, these results suggest that changes in BM protein levels correlate with pNF growth and treatment response. Thus, determining BM protein levels in pNF could have potential for diagnosis and treatment response monitoring.

### The MAPK pathway mediates TGF-β1–induced BM protein expression in Schwann cells.

**Figure 7:**
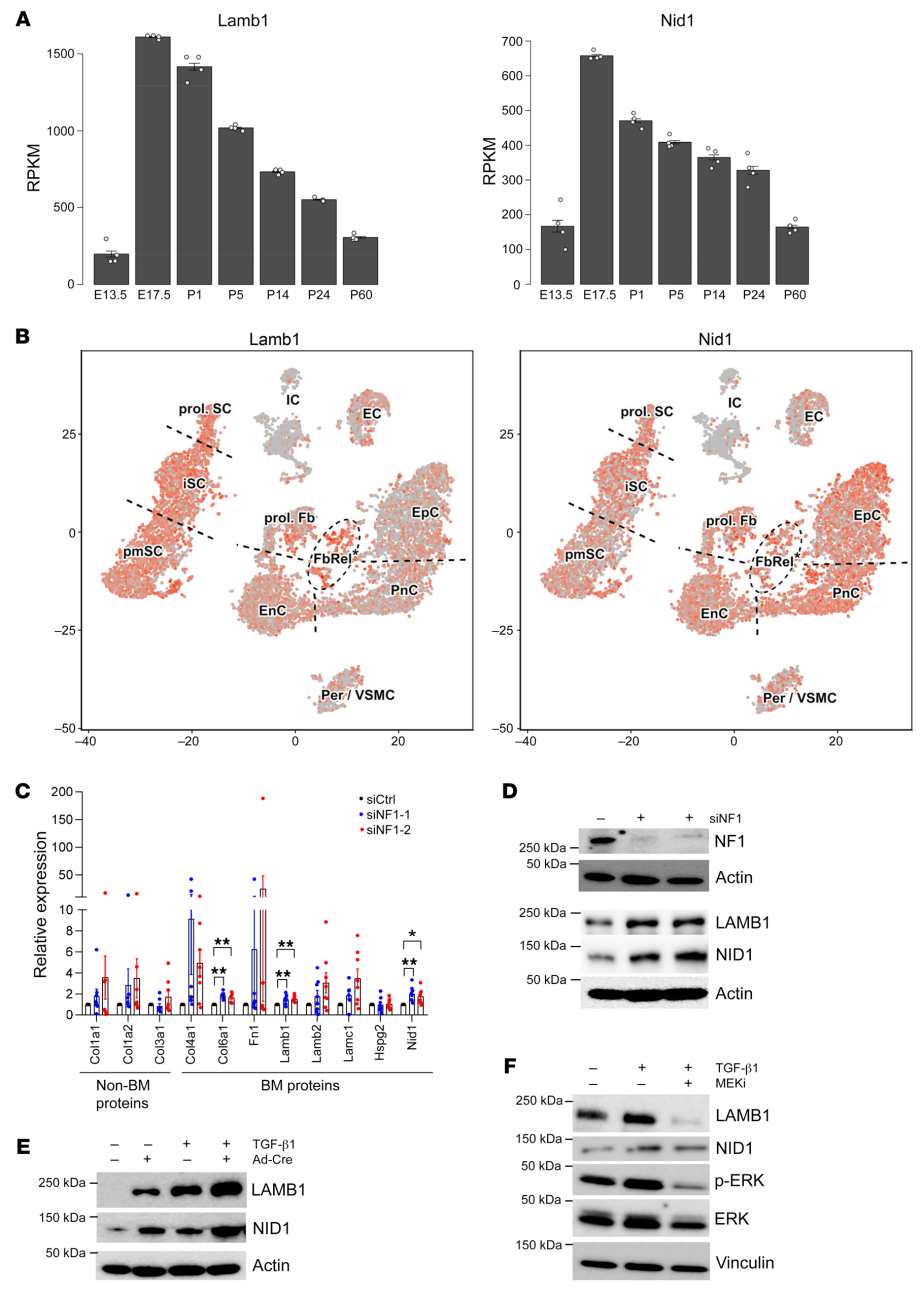
The MAPK pathway mediates TGF-β1–induced BM protein expression in Schwann cells. (**A**) Bulk RNA-Seq results showing the mRNA levels of *Lamb1* and *Nid1* on a time scale of sciatic nerve development (generated from the Sciatic Nerve Atlas; https://snat.ethz.ch). RPKM, reads per kilobase of transcript per million mapped reads. (**B**) T-distributed stochastic neighbor embedding (t-SNE) plots showing *Lamb1* and *Nid1* expression in sciatic nerve cells collected from mice at P1 (generated from the Sciatic Nerve Atlas). EC, endothelial cells; EnC, endoneurial cells; EpC, epineurial cells; FbRel, fibroblast-related cells; IC, immune cells; iSC, immature Schwann cells; Per/VSMC, pericyte and vascular smooth muscle cells; pmSC, promyelinating Schwann cells; PnC, perineurial cells; prol. Fb, proliferating fibroblast-like cells; prol. SC, proliferating Schwann cells. (**C**) Quantitative PCR analysis showing the mRNA levels of the indicated BM and non-BM ECM proteins in hTERT ipn02.3 2λ cells after *NF1* knockdown (*n* = 8). (**D**) Representative Western blots showing the expression levels of the indicated proteins in hTERT ipn02.3 2λ cells after *NF1* knockdown. *n* = 3. (**E**) Representative Western blots showing the expression levels of the indicated proteins in *Nf1^fl/fl^* E13.5 DRG neurosphere cells following transduction with adenovirus-Cre and/or treatment with TGF-β1. *n* = 3. (**F**) Representative Western blots showing the expression levels of the indicated proteins in hTERT ipNF05.5 cells after treatment with TGF-β1 alone or TGF-β1 plus MEKi. *n* = 3. Data are shown as means ± SEM. Comparisons among groups were performed by Dunnett’s test. *P < 0.05, **P < 0.01.

To begin to understand the cellular and molecular mechanisms underlying Schwann cell deposition of BM proteins in pNF, we searched the Sciatic Nerve Atlas database (https://snat.ethz.ch/index.html) for the expression pattern of LAMB1 and NID1 during mouse sciatic nerve development. Based on bulk RNA-Seq with tissues collected on a time scale of sciatic nerve development, both LAMB1 and NID1 are abundantly expressed at E17.5 when immature Schwann cells (iSCs) form and dominate during Schwann cell differentiation (Figure 7A). Moreover, the scRNA-Seq from P1 sciatic nerve demonstrates that both LAMB1 and NID1 are abundantly expressed by iSCs and promyelinating Schwann cells (Figure 7B). These data correlate with a large body of evidence that the cells of origin of pNF reside in the earlier stages of Schwann cell lineage (4).

In addition to the cellular association with pNF tumorigenesis, we asked whether these BM proteins are molecularly associated with pNF pathogenesis by examining their expression levels in the human Schwann cell line hTERT ipn02.3 2λ after siRNA-mediated *NF1* knockdown. The mRNA levels of selective BM components, including COL6A1, LAMB1, and NID1, were upregulated in cells with *NF1* knockdown (Figure 7C). We confirmed the upregulation of LAMB1 and NID1 by Western blots (Figure 7D). We also verified the regulation of LAMB1 and NID1 in *Nf1^fl/fl^* E13.5 DRG neurosphere cells: upon adenovirus-Cre–mediated *Nf1* loss (Supplemental Figure 7), the expression levels of LAMB1 and NID1 were increased. Importantly, this upregulation was further increased by TGF-β1 treatment (Figure 7E). We also confirmed the upregulation of LAMB1 and NID1 by TGF-β1 in the human pNF cell line hTERT ipNF05.5 (Figure 7F). Notably, the expression level of phospho-ERK was increased by TGF-β1 treatment in hTERT ipNF05.5 cells (Figure 7F), consistent with the upregulation of phospho-ERK observed in TGF-β1 capsule–implanted mice (Figure 3F), suggesting activation of the MAPK signaling pathway by TGF-β1 in pNF. Furthermore, the induction of LAMB1 and NID1 by TGF-β1 was attenuated by MEKi treatment (Figure 7F), indicating that the MAPK pathway mediates TGF-β1–induced LAMB1 and NID1 expression. Altogether, these data support the existence of a regulatory pathway in pNF whereby *NF1* loss (an intrinsic factor) and TGF-β1 signaling (an extrinsic factor) cause increased deposition of BM proteins.

## Discussion

In this study, we investigated ECM dynamics in pNF using both proteomic and transcriptomic approaches. We systematically characterized ECM profiles during pNF development and further demonstrated that MEK inhibitor (MEKi) can reduce ECM deposition, which likely plays a role in its therapeutic efficacy. Importantly, compared with seemingly abundant collagen isoforms, BM proteins were shown to contribute significantly to ECM accumulation and decrease in response to MEKi treatment. Mechanistically, we established a regulatory network for BM protein deposition by linking *NF1* loss with TGF-β1 regulation of BM deposition by Schwann cells. Together, these data provide insight into the regulation underlying ECM dynamics in pNF during tumorigenesis and treatment response. However, these results only provide a “snapshot” of ECM composition and cellular interaction in pNF development and treatment response at the specific time points analyzed. Future studies of different disease stages will be needed to capture the evolution of cellular composition and ECM remodeling.

ECM enrichment is a salient characteristic of the pNF microenvironment; it has been reported that more than 50% of neurofibroma dry weight is collagen (27). Through immunohistochemistry and in situ hybridization studies, different isoforms of collagen were identified in neurofibromas, including collagen types I, III, IV, V, and VI (19, 27–30). These results offered important insights into the ECM profiles of pNF. However, what constitutes the ECM in pNF has not been completely elucidated. In this study, we used mass spectrometry to perform a systematic, quantitative, and unbiased investigation of the ECM profile in pNF. Surprisingly, compared with WT, the collagen profile was not dramatically changed during pNF development (Figure 1F), likely because of the lack of classic profibrogenic myofibroblasts (16). This is direct evidence systematically outlining the collagen profile in pNF, resolving a long-standing question about the distribution and nature of collagens in pNF.

Comparatively, some BM proteins are significantly upregulated in pNF, and they are indeed the most enriched ECM proteins in pNF (Figure 4A and Table 1). This quantitative analysis confirmed previous immunohistochemistry studies showing that BM protein accumulation is a striking feature of pNF ECM (19). It is also consistent with a recent publication reporting that the genes involved in the interaction between basal lamina, a portion of BM, and β_1_ integrin were broadly upregulated in pNF based on scRNA-Seq analysis (25). Notably, BM protein enrichment is intimately associated with pNF pathogenesis, as *Nf1* knockdown in Schwann cells increases BM protein production. Furthermore, the overexpression of BM proteins may play an important role in pNF development, as BM proteins are known to have key signaling roles in regulating cell polarity, differentiation, and tissue maintenance throughout development as well as in adult tissues and organs (31). For instance, laminin-1 was reported to direct mammary-specific gene transcription in mouse mammary epithelial cells (32). Moreover, multiple lines of evidence suggested that BM proteins are intimately linked to cancer progression. High NID1 expression correlates with disease progression in several types of cancers (33–35). LAMB1 is overexpressed in capillary BMs in breast tumor tissues, which is associated with breast cancer progression and metastasis (36). As for the possible mechanisms by which BM proteins promote tumor progression in pNF, in addition to their signaling roles, an interesting and relevant hypothesis is that the overexpression of selective BM proteins may create a foreign BM and/or impair the natural BM ultrastructure, leading to the dissociation between neuron and Schwann cells, which has been frequently reported in pNF tumorigenesis (3, 37–41). Taken together, our mass spectrometry results reveal a unique ECM signature highlighting BM proteins over collagens in pNF. Further investigation of the role of BM proteins in pNF is warranted.

Fibroblasts are well known as the principal cell type that deposits ECM throughout the body under both normal physiological and pathological conditions (15). Regarding pNF, a fundamental question in the field is whether and how neoplastic Schwann cells contribute to ECM accumulation in addition to fibroblasts. Using mass spectrometry coupled with scRNA-Seq, we provide definitive evidence that neoplastic Schwann cells in pNF secrete multiple BM proteins, confirming previous studies with immunohistochemistry and Northern blot analysis for several BM proteins (19, 42). Mechanistically, as a master regulator of fibrosis, TGF-β1 regulates the expression of different ECM proteins, including collagens and BM proteins (43, 44). TGF-β1 capsule implantation promotes growth of pNF, which can be plausibly attributed to the stimulation of ECM deposition. We reason that TGF-β1 acts as a modifier for pNF growth and ECM deposition, and targeting TGF-β1 is a promising strategy for pNF treatment. However, direct targeting of the TGF-β1 signaling pathway is therapeutically challenging owing to the multifaceted roles of TGF-β1 in different cellular contexts. In addition to ECM deposition by fibroblasts, TGF-β1 acts as a tumor suppressor because of its inhibition of epithelial cell proliferation. TGF-β1 can also promote tumor immune evasion by conferring an immunosuppressive phenotype to T cells (45–47). Therefore, this mixed regulation on different cell types could plausibly be relevant to the modest therapeutic efficacy of pirfenidone, an antifibrotic reagent known to attenuate cellular response to TGF-β1 (48). Although direct targeting of TGF-β1 for fibrosis inhibition remains to be explored in pNF, indirect targeting may be a more feasible strategy for TGF-β1–induced fibrosis in that the profibrotic effects of TGF-β1 are regulated by interactions with many other signaling pathways, including the ERK/MAPK pathway (49). Here, using mass spectrometry, we show that MEK inhibition decreases ECM deposition (Figure 2), which is in line with the inhibition of fibrosis observed upon MEKi treatment in kidney fibrosis (50).

It has previously been shown that macrophages and T cells are abundant in pNF and contribute to neurofibroma development by causing local inflammation (14, 51). Our study reveals a novel function for how macrophages and T cells contribute to neurofibroma pathogenesis: we have found that TGF-β1 is predominantly expressed by macrophages and T cells in the pNF tumor microenvironment. This TGF-β1 then signals to Schwann cells and fibroblasts to express and secrete BM proteins, key components of the pNF ECM that are downregulated in response to MEKi treatment. Our studies therefore delineate the cellular and molecular regulation of the deposition of ECM proteins, specifically BM proteins, in the contexts of pNF tumor development and treatment response. These findings reveal a new potential mechanism by which MEKi inhibits pNF growth and identify a new avenue for therapeutic investigation.

## Methods

### Mice.

The *H7;Nf1mut* mice have been described previously (10). Mice were maintained in a clean mouse facility at University of Texas Southwestern Medical Center. All procedures involving animal maintenance and experimental use were performed following the guidelines approved by the Institutional Animal Care and Use Committee at University of Texas Southwestern Medical Center.

### Cell culture.

Isolation and culture of E13.5 DRG neurosphere cells were performed as previously described (6, 9, 52). For isolation and culture of primary Schwann cells and fibroblasts, pNF DRGs were first collected and digested with a cocktail of 2 mg/mL collagenase and 1.5 mg/mL dispase at 37°C for 30 minutes. Debris was removed with a 40 μm strainer. A pre-plating procedure was used to separate fibroblasts from Schwann cells. Briefly, the cell mixture suspension was plated in cell culture plates for 1 hour to allow fibroblasts to attach to the plates. Then the unattached cells were collected and cultured in E13.5 DRG neurosphere cell culture medium to enrich for neoplastic Schwann cells. Primary fibroblasts were cultured in DMEM supplemented with 10% FBS and 1% penicillin-streptomycin. The human pNF cell line hTERT ipNF05.5 (ATCC, CRL-3388) and the normal Schwann cell line hTERT ipn02.3 2λ (ATCC, CRL-3392) were cultured in DMEM supplemented with 10% FBS and 1% penicillin-streptomycin. All cells were cultured at 37°C under 5% CO_2_. For the TGF-β1 treatment, cells were incubated with 10 ng/mL TGF-β1 for 72 hours. For MEKi treatment, 1 μg/mL PD0325901 (Selleckchem, S1036) was added to cell culture for 72 hours.

### DRG ex vivo culture.

Whole spinal cords were extracted immediately after euthanasia (4, 10). Paraspinal pNF DRGs were excised and transferred to ice-cold DMEM. DRGs were cultured in the E13.5 DRG neurosphere cell growth medium. For MEKi treatment, 1 μg/mL PD0325901 was added to culture medium for 72 hours.

### In vivo TGF-β1 studies.

Recombinant human TGF-β1 (PeproTech, 100-21) was solubilized in PBS and dosed to 100 μg per kg body weight for 4 weeks. TGF-β1 or vehicle was subcutaneously delivered by implantable osmotic minipump (RWD Life Science, 1004w).

### DRG size quantification.

Whole spinal cord dissection was performed as previously described (4, 10), followed by image capture. The length and width of DRGs were measured by ImageJ (NIH). The volume of each DRG was calculated by the formula length × width^2^ × 0.5.

### Mass spectrometry.

Mouse tissues were collected and lysed with the CNMCS Compartmental Protein Extraction Kit (Millipore, 2145) according to the manufacturer’s protocol. The final pellets were then sent to the Proteomics Core at University of Texas Southwestern Medical Center. Data were acquired by the facility core and analyzed using Proteome Discoverer 2.4 (Thermo Fisher Scientific), followed by searching of the mouse protein database from UniProt. For data analysis, to increase the level of confidence for the identified proteins, the results were filtered with a threshold of 10 peptide-spectrum matches (PSMs). All the protein with more than 10 PSMs was added up and calculated as total protein abundance for WT and pNF. For each individual target, the abundance ratio was calculated by its abundance normalized to total protein abundance (abundance ratio of protein X = protein X abundance / total protein abundance). This abundance ratio for each protein was used to compare between WT and pNF. Student’s *t* test was used to calculate statistical difference. *P* less than 0.05 was considered to be statistically significant.

### ScRNA-Seq.

Paraspinal pNF DRGs were excised immediately after euthanasia and minced into pieces, followed by digestion with a cocktail of type I collagenase (2.5 mg/mL) and dispase B (2.5 mg/mL) for 20 minutes. Debris was removed using a 40 μm cell strainer, and the cell suspension was centrifuged at 200*g*. Cell pellets were resuspended in 100 μL of 1× PBS/0.04% BSA. Cell viability was checked with the TC20 Automated Cell Counter (Bio-Rad). Cells were processed in the Chromium Single Cell Gene Expression (10x Genomics) to build the 3′ transcriptome libraries and sequenced in the Next Generation Sequencing Core at University of Texas Southwestern Medical Center.

### Analyses of single-cell transcriptomes.

Cell Ranger 5.0.1 (10x Genomics, https://www.10xgenomics.com/) was used to process the raw sequencing data. BCL files were converted to FASTQ files and aligned to mouse (mm10) reference transcriptome. Transcript counts of each cell were quantified using unique molecular identifier and valid cell barcode. The gene expression matrix from Cell Ranger was used as input to the Seurat R package (v4.0.2) for downstream analysis (53). Cells with fewer than 200 genes per cell and high mitochondrial gene content were filtered out. The global-scaling normalization method LogNormalize was used for normalization. A subset of genes exhibiting high variation across the single cells was determined. The highly variable genes were calculated using the FindVariableFeatures module. The Shared Nearest Neighbor (SNN) graph was constructed with the FindNeighbors module by determination of the k-nearest neighbors of each cell. The clusters were then identified by optimization of SNN modularity using the FindClusters module. We obtained 14 clusters with a resolution of 0.4. Clusters were named on the basis of known gene markers specific to various cell types. Differential expression analysis, clusters visualization, and plotting were all performed with Seurat.

### Protein extraction and Western blots.

Protein was extracted with RIPA buffer (Thermo Fisher Scientific, 89901). Protein concentration was determined by Pierce BCA protein assay kit (Thermo Fisher Scientific, PI23225). Protein was separated by SDS-PAGE and transferred to PVDF membranes. Membranes were blocked with 5% milk for 1 hour, followed by incubation with primary antibodies at 4°C overnight. Then membranes were incubated with HRP-conjugated secondary antibodies for 2 hours. The ChemiDoc Touch Imaging System (Bio-Rad) was used to capture the images. The antibodies used for Western blots are listed in Supplemental Table 1.

### H&E and immunohistochemistry and immunofluorescence staining.

Mouse tissues were fixed in 10% formalin at room temperature and embedded into paraffin. Paraffin blocks were cut into 5 μm slides. Slides were deparaffinized and rehydrated, followed by H&E staining. For immunohistochemistry staining, slides were incubated in TE buffer (0.01 M Tris, 0.05% Tween-20, 1 mM EDTA, pH 9.0) and heated for 20 minutes to retrieve antigens. Tissue slides were blocked in 10% donkey serum and incubated with primary antibodies diluted in 3% donkey serum at 4°C overnight, followed by incubation with secondary antibodies coupled to biotin at room temperature for 1 hour. Slides were subsequently incubated with avidin-biotinylated peroxidase conjugate according to VECTASTAIN Elite ABC kit (Vector Laboratories) procedure, and visualized by addition of the DAB substrate. Finally, slides were counterstained with hematoxylin and coverslipped. For immunofluorescence staining, after antigen retrieval, slides were blocked in 10% goat serum and incubated with primary antibodies at 4°C overnight. Then slides were incubated with secondary antibodies at room temperature for 1 hour and mounted with the mounting medium. The antibodies used for immunohistochemistry and immunofluorescence experiments are listed in Supplemental Table 1.

### NF1 siRNA transfection.

Two predesigned siRNAs against human *NF1* were purchased from MilliporeSigma (SASI_Hs01_00129008 and SASI_Hs01_00129006). Transfection was performed with Lipofectamine RNAiMAX Transfection Reagent following standard protocol (Thermo Fisher Scientific, 13778030). After 72 hours, cells were collected and analyzed.

### RNA extraction, cDNA synthesis, and quantitative PCR.

RNA was extracted with Qiagen RNeasy Mini Kit (Thermo Fisher Scientific, NC9307831), followed by reverse transcription to cDNA with iScript cDNA Synthesis Kit (Bio-Rad, 1708891BUN). Quantitative PCR (qPCR) was performed with a QuantStudio 3 machine (Applied Biosystems). For qPCR result analysis, the 2^−ΔΔCt^ method was used to calculate the fold change. Primers are listed in Supplemental Table 2.

### Data deposition.

ScRNA-Seq data were deposited in the NCBI’s Gene Expression Omnibus database (GEO GSE227456).

### Statistics.

Data are shown as mean ± SEM. *P* values were calculated by 2-tailed Student’s *t* test, unless otherwise specified. *P* less than 0.05 was considered to be statistically significant.

### Study approval.

Animal care and use in this study were approved by the Institutional Animal Care and Use Committee at University of Texas Southwestern Medical Center.

## Author contributions

CJ and LQL conceived and designed the study. CJ and LQL developed methodology. CJ, AK, ZY, TS, and YW acquired data (and provided animals, generated cells and reagents, provided facilities, etc.). CJ, AK, ZY, RMM, CX, and LQL analyzed and interpreted data (e.g., statistical analysis, biostatistics, computational analysis). CJ, AK, ZY, RMM, CX, and LQL wrote, reviewed, and/or revised the manuscript. CJ and LQL provided administrative, technical, or material support (i.e., reporting or organizing data, constructing databases). LQL supervised the study.

## Supplementary Material

Supplemental data

## Figures and Tables

**Table 1:**
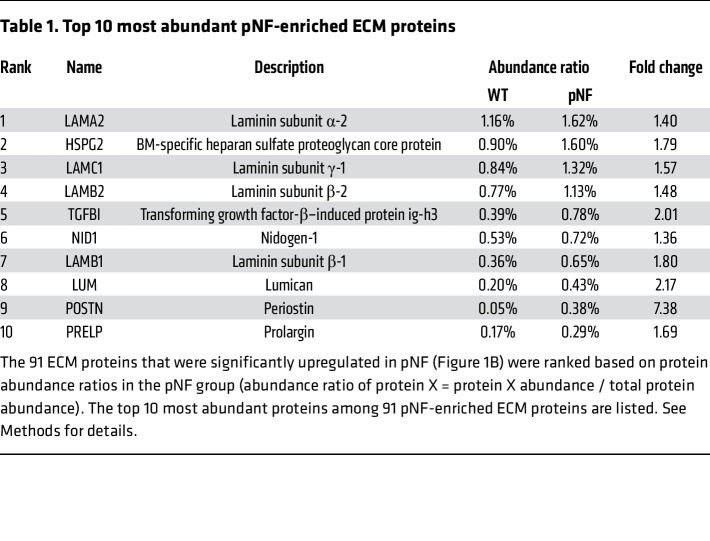
Top 10 most abundant pNF-enriched ECM proteins

## Version 1 05/04/2023

In-Press Preview

## Version 2 06/15/2023

Electronic publication
