# Supplementary material for: Basement membrane proteins in extracellular matrix characterize NF1 neurofibroma development and response to MEK inhibitor
Source: J Clin Invest. 2023 Jun 15;133(12):e168227. doi: 10.1172/JCI168227 (PMC10266775; doi:10.1172/JCI168227)
Supplement: Supplemental data [file jci-133-168227-s105.pdf]

Supplemental Figures and Tables for:

Basement membrane proteins in extracellular matrix characterize NF1 neurofibroma development and response to MEK inhibitor

Chunhui Jiang[1], Ashwani Kumar[2], Ze Yu[2], Tracey Shipman[1], Yong Wang[1], Renee M. McKay[1], Chao Xing[2,3], and Lu Q. Le[1,4,5,6,7]

[1]Department of Dermatology, [2]Eugene McDermott Center for Human Growth and Development, [3]Lyda Hill Department of Bioinformatics, [4]Simmons Comprehensive Cancer Center, [5]UTSW Comprehensive Neurofibromatosis Clinic, [6]Hamon Center for Regenerative Science and Medicine, [7]O'Donnell Brain Institute, University of Texas Southwestern Medical Center at Dallas, Dallas, Texas, 75390-9069, USA

[*]Corresponding Author:

Lu Q. Le, M.D., Ph.D.
Professor of Dermatology
Hamon Center for Regenerative Science and Medicine
Simmons Comprehensive Cancer Center
O'Donnell Brain Institute
University of Texas Southwestern Medical Center
5323 Harry Hines Blvd
Dallas, TX 75390-9069
Phone: (214) 648-5781
Fax: (214) 648-5553
E-mail: Lu.Le@UTSouthwestern.edu

Running title: Basement membrane proteins in neurofibroma growth and treatment response

Keywords: plexiform neurofibroma, extracellular matrix, basement membrane, TGF-β1, macrophages, mass spectrometry, single-cell RNA-sequencing

The authors declare no potential conflicts of interest.

A

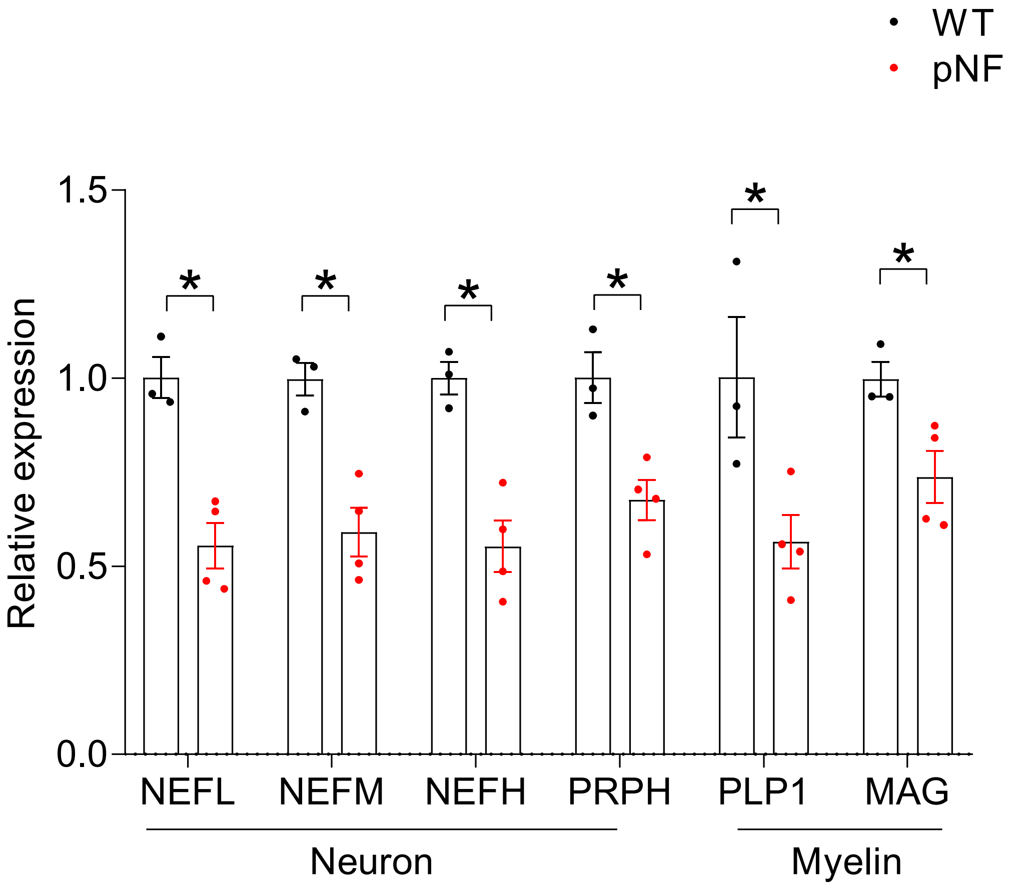
WT
pNF
1.5
1.0
0.5
0.0
Relative expression
NEFL
NEFM
NEFH
PRPH
PLP1
MAG
Neuron
Myelin
*

B

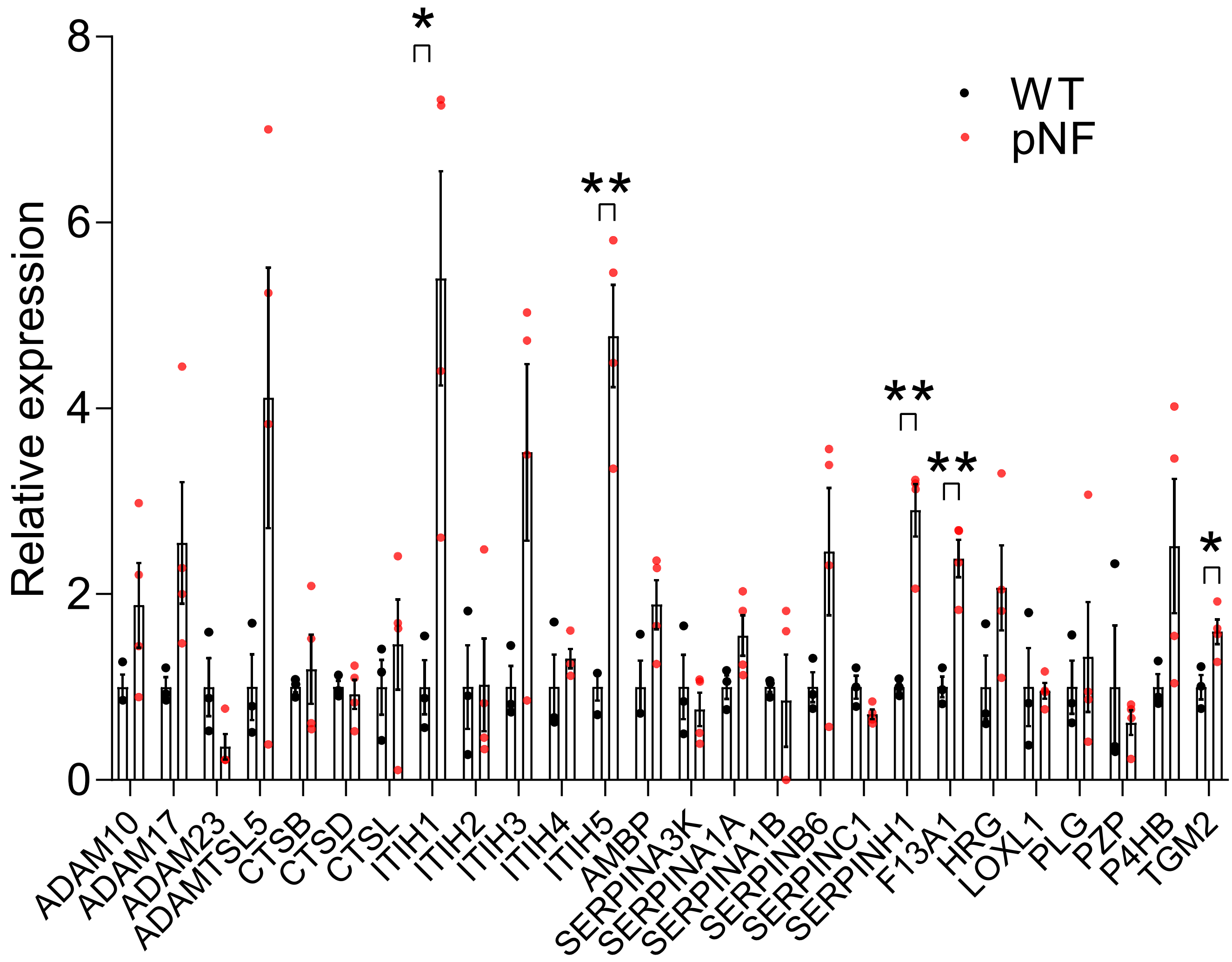
WT
pNF
8
6
4
2
0
Relative expression
ADAM10
ADAM17
ADAM23
ADAMTSL5
CTSB
CTSD
CTSL
ITIH1
ITIH2
ITIH3
ITIH4
ITIH5
AMBP
SERPINA3K
SERPINA1A
SERPINA1B
SERPINB6
SERPINC1
SERPINH1
F13A1
HRG
LOXL1
PLG
PZP
P4HB
TGM2
*
**

Supplemental Figure 1. Mass spectrometry data analysis between WT and pNF. (A) Mass spectrometry data reveals down-regulation of neuron- and myelination-related targets in pNF based on abundance ratios. NEFL, neurofilament light chain; NEFM, neurofilament medium chain; NEFH, neurofilament heavy chain; PRPH, peripherin; PLP1, proteolipid protein 1; MAG, myelin-associated glycoprotein. **(B)** Mass spectrometry data reveals up-regulation of several ECM regulators in pNF based on abundance ratios. Data are shown as the means ± s.e.m. Comparisons among groups were performed by student's t test. * $P< 0.05$. ** $P< 0.01$.

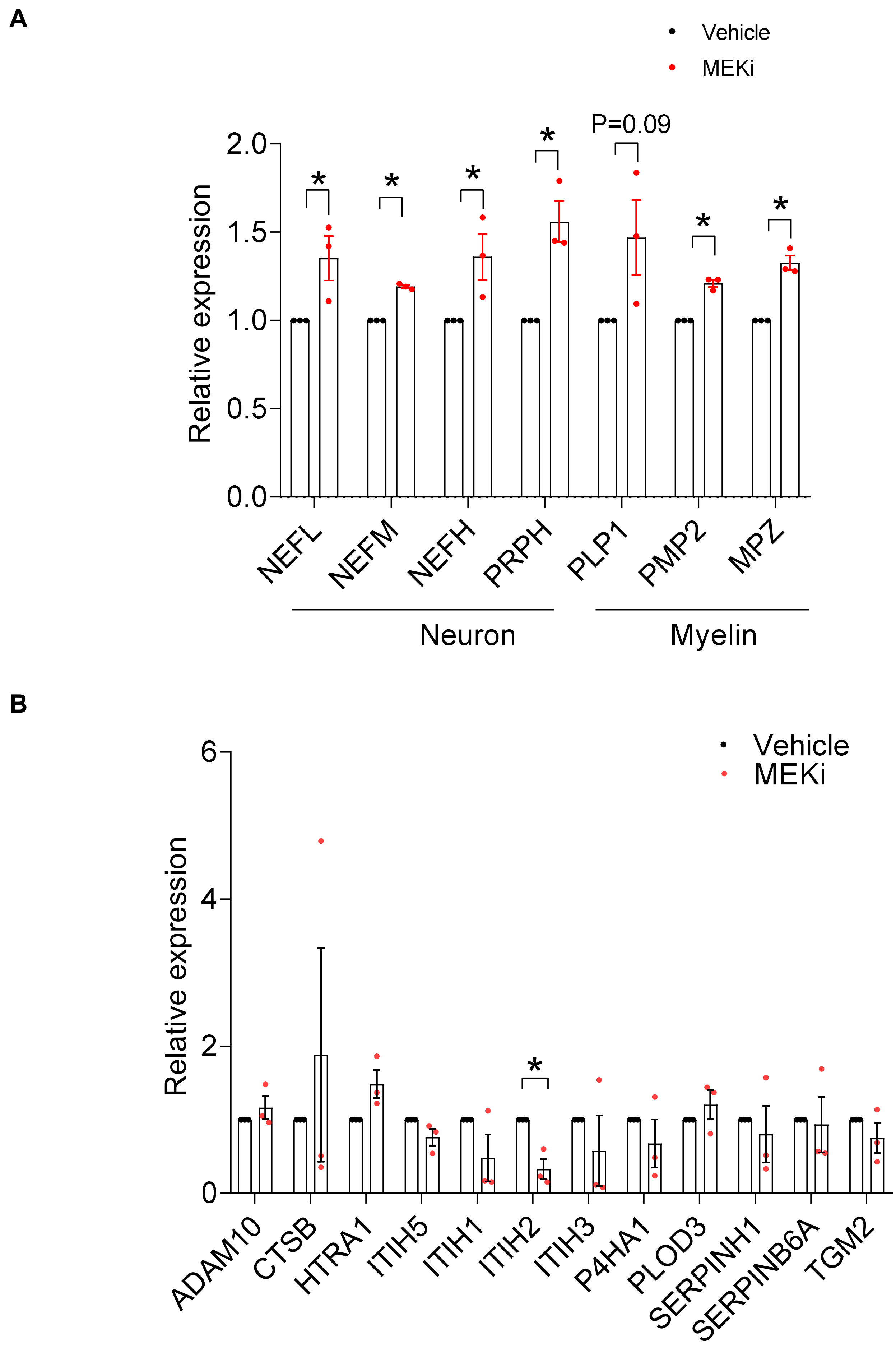
A
Vehicle
MEKi
P=0.09
*
Relative expression
2.0
1.5
1.0
0.5
0.0
NEFL
NEFM
NEFH
PRPH
PLP1
PMP2
MPZ
Neuron
Myelin
B
Vehicle
MEKi
6
4
2
0
Relative expression
ADAM10
CTSB
HTRA1
ITIH5
ITIH1
ITIH2
ITIH3
P4HA1
PLOD3
SERPINH1
SERPINB6A
TGM2

Supplemental Figure 2. Mass spectrometry data analysis between MEKi and Vehicle treated pNF DRGs. (**A**) Mass spectrometry data reveals up-regulation of neuron- and myelination-related targets in MEKi-treated group based on abundance ratios. NEFL, neurofilament light chain; NEFM, neurofilament medium chain; NEFH, neurofilament heavy chain; PRPH, peripherin; PLP1, proteolipid protein 1; MAG, myelin-associated glycoprotein. (**B**) Mass spectrometry data reveals expression of ECM regulators between MEKi and Vehicle treated pNF DRGs. Data are shown as the means ± s.e.m. Comparisons among groups were performed by student's t test. * $P < 0.05$.

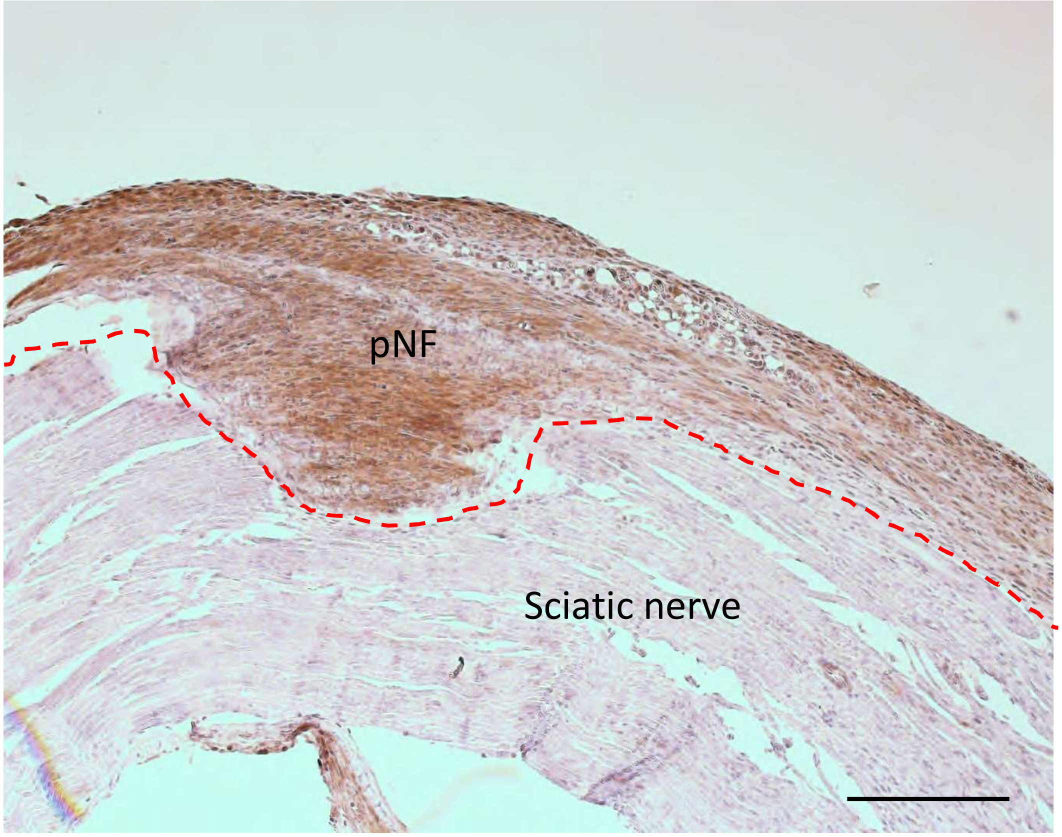
pNF
Sciatic nerve

Supplemental Figure 3. High expression of TGF-β1 in pNF compared to the normal sciatic nerve region. Representative immunohistochemistry image showing the expression of TGF-β1 in the sciatic nerve of a nude mouse transplanted with E13.5 DRG neurosphere cells. The dashed red line indicates the tumor-nerve boundary. Scale bar, 200 μm.

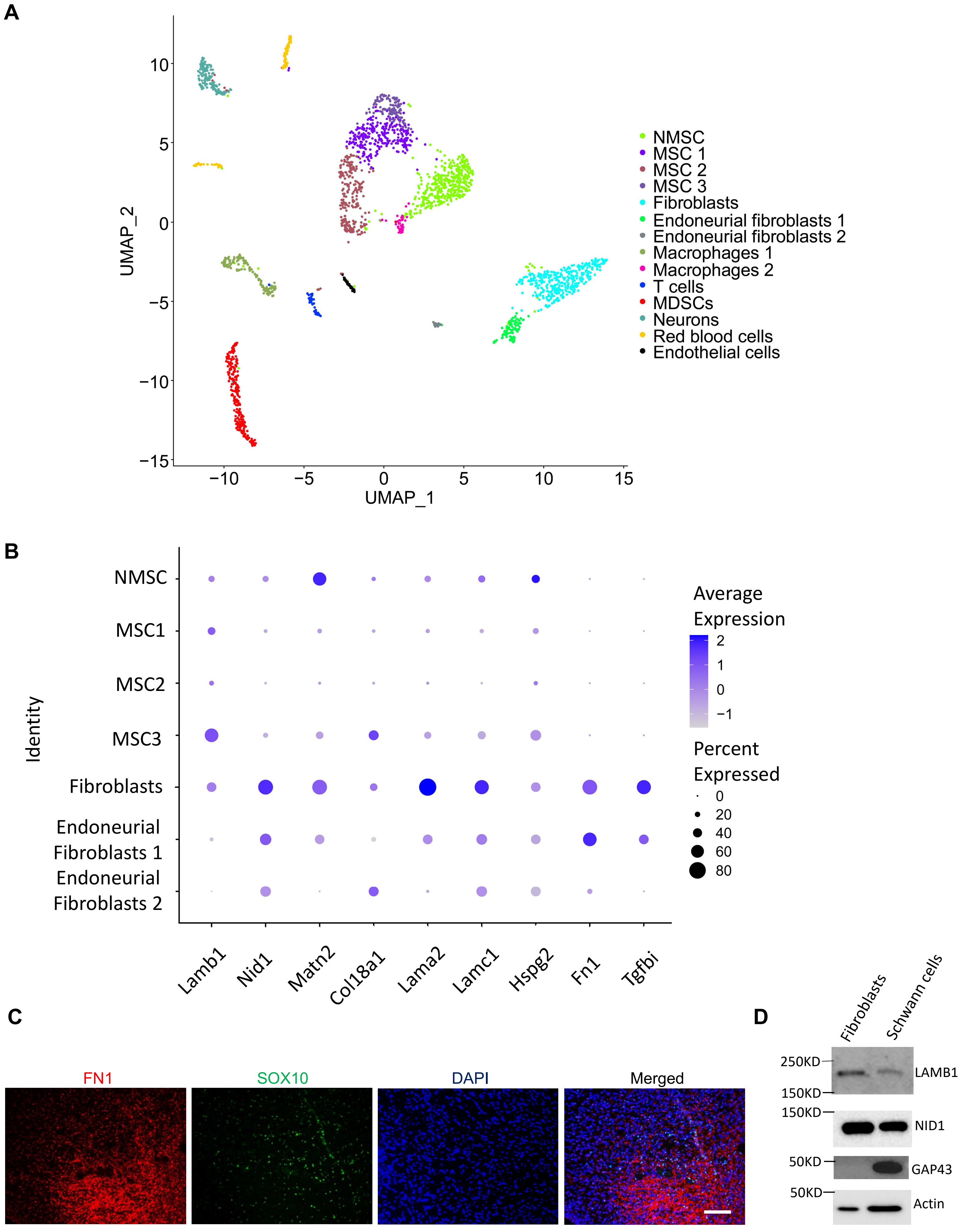
A
UMAP_2
UMAP_1
NMSC
MSC 1
MSC 2
MSC 3
Fibroblasts
Endoneurial fibroblasts 1
Endoneurial fibroblasts 2
Macrophages 1
Macrophages 2
T cells
MDSCs
Neurons
Red blood cells
Endothelial cells
B
Identity
NMSC
MSC1
MSC2
MSC3
Fibroblasts
Endoneurial Fibroblasts 1
Endoneurial Fibroblasts 2
Lamb1
Nid1
Matn2
Col18a1
Lama2
Lamc1
Hspg2
Fn1
Tgfbi
Average Expression
Percent Expressed
C
FN1
SOX10
DAPI
Merged
D
Fibroblasts
Schwann cells
250KD
150KD
LAMB1
150KD
NID1
50KD
GAP43
50KD
Actin

Supplemental Figure 4. Sc RNA-seq analysis verified by biochemical experiments. (**A**) A Uniform Manifold Approximation and Projection (UMAP) plot showing the cell populations identified by scRNA-Seq. NMSC, non-myelinating Schwann cells; MSC, myelinating Schwann cells; MDSC, myeloid-derived suppressor cells. (**B**) Dot plot showing the expression of indicated BM proteins in Schwann cells and fibroblasts. (**C**) Immunofluorescence images showing co-immunofluorescence of FN1 and SOX10 in mouse pNF tissue. Scale bar, 100 μm. (**D**) Representative western blots showing the expression of LAMB1 and NID1 in primary fibroblasts and Schwann cells. GAP43 was used as a marker for Schwann cells.

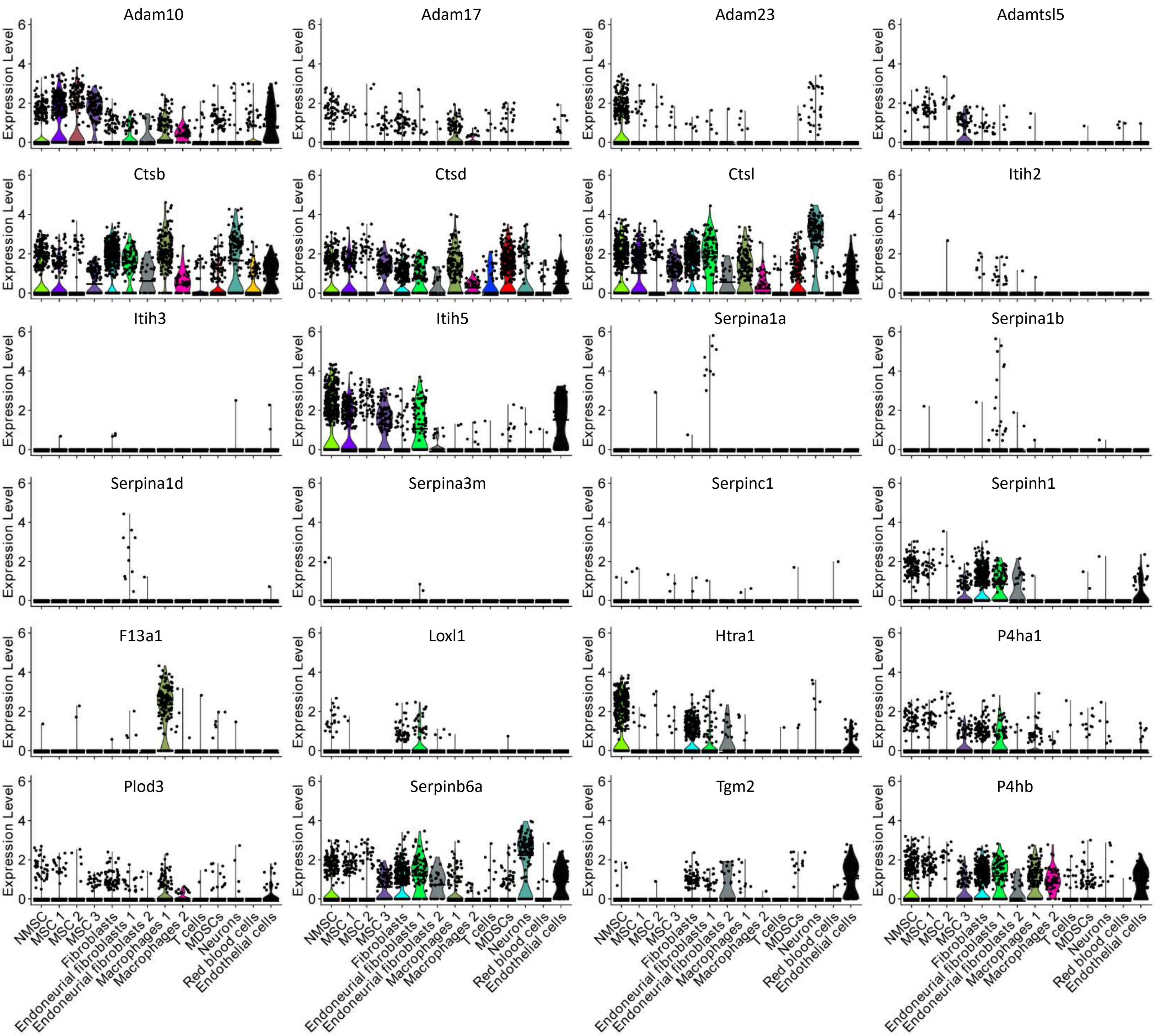
Adam10
Adam17
Adam23
Adamtsl5
Ctsb
Ctsd
Ctsl
Itih2
Itih3
Itih5
Serpina1a
Serpina1b
Serpina1d
Serpina3m
Serpinc1
Serpinh1
F13a1
Loxl1
Htra1
P4ha1
Plod3
Serpinb6a
Tgm2
P4hb
Expression Level
0
2
4
6
NMSC
MSC 1
MSC 2
MSC 3
Fibroblasts
Endoneurial fibroblasts 1
Endoneurial fibroblasts 2
Macrophages 1
Macrophages 2
T cells
MDSCs
Neurons
Red blood cells
Endothelial cells

Supplemental Figure 5. Expression of ECM regulators in pNF. Violin plots showing the expression of indicated ECM regulators in different cell populations. NMSC, non-myelinating Schwann cells; MSC, myelinating Schwann cells; MDSC, myeloid-derived suppressor cells.

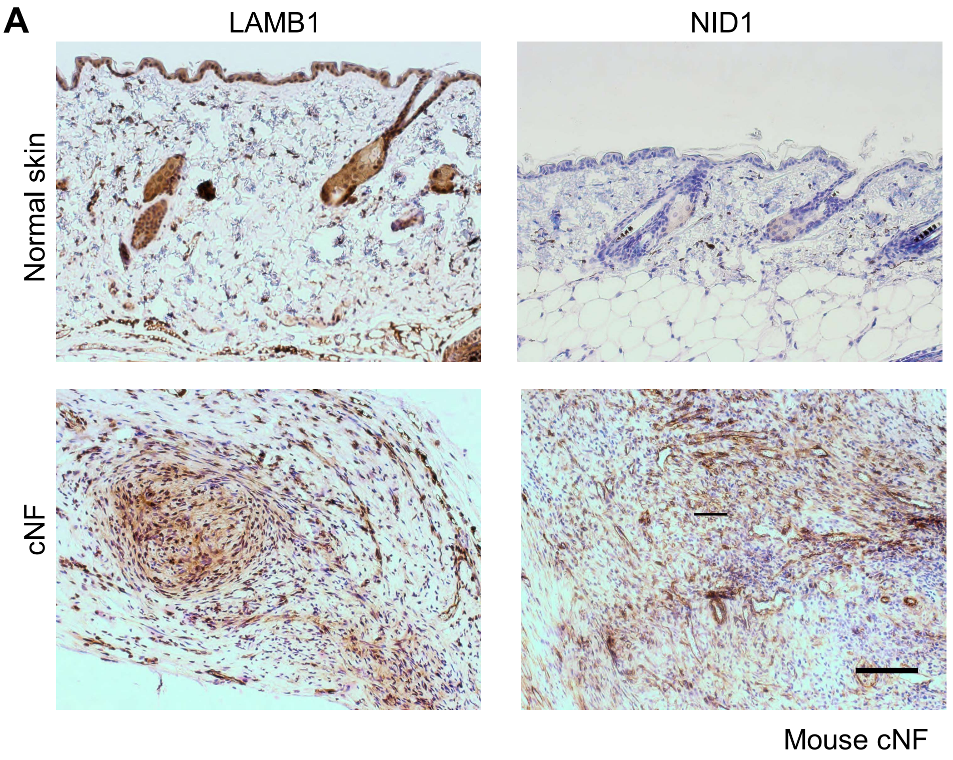
A
LAMB1
NID1
Normal skin
cNF
Mouse cNF

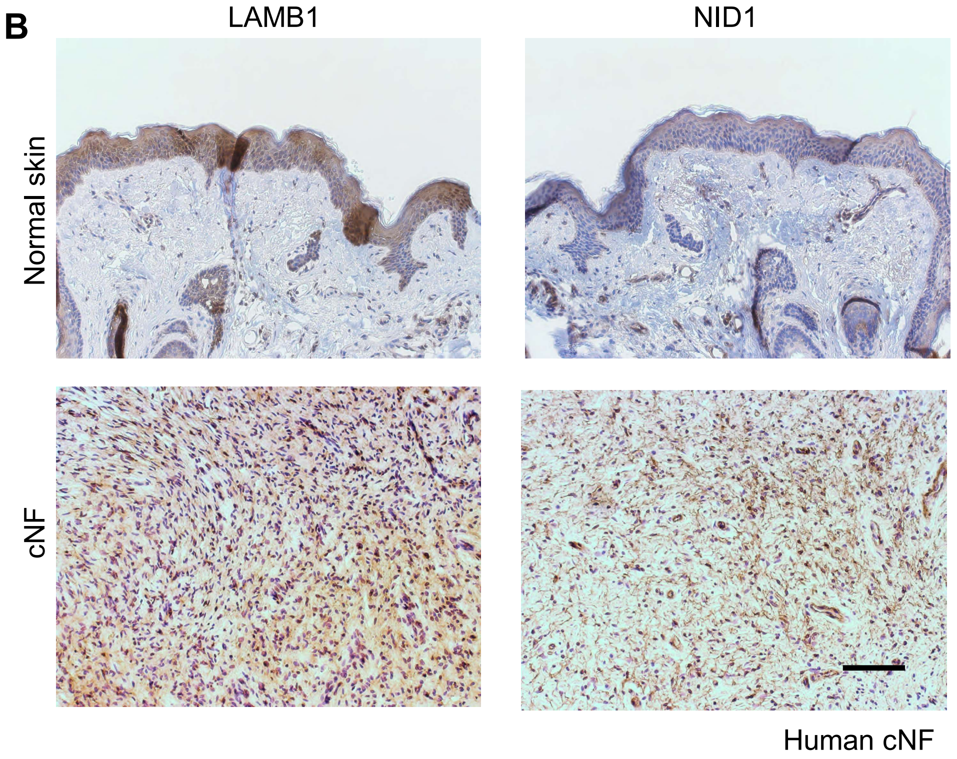
B
LAMB1
NID1
Normal skin
cNF
Human cNF

Supplemental Figure 6. BM protein expression in mouse and human cNF. (**A**) Representative immunohistochemistry image showing the expression of LAMB1 and NID1 in mouse cNF and normal skin. Scale bar, 100 μm. (B) Representative immunohistochemistry image showing the expression of LAMB1 and NID1 in human cNF and normal skin. Scale bar, 100 μm.

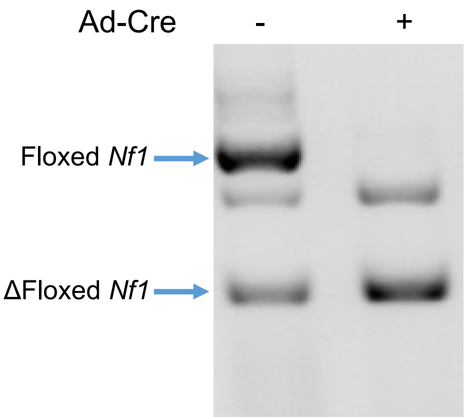
Ad-Cre
-
+
Floxed *Nf1*
ΔFloxed *Nf1*

Supplemental Figure 7. Deletion of *Nf1* in *Nf1*$^{f/f}$ E13.5 DRGs following transduction with adenovirus-Cre. A representative genotyping agarose gel image showing the loss of *Nf1* in *Nf1*$^{f/f}$ E13.5 DRG neurosphere cells after transduction with adenovirus-Cre.

Antibody	Company	Identifier
Rabbit anti-LAMB1	Thermo Fisher	PA5-27271
Rabbit anti-NID1	Abcam	ab254325
Rabbit anti-p-ERK	Cell Signaling	4370L
Rabbit anti-ERK	Cell Signaling	4695S
Mouse anti-Actin	Sigma	A5441
Rabbit anti-Vinculin	Abcam	ab91459
Rabbit anti-TGF-β1	Novus Biologicals	NBP1-03276
Mouse anti-TGF-β1	Santa Cruz	sc-130348
Rabbit anti-S100 β	Abcam	ab52642
Rabbit anti-SOX10	Abcam	ab180862
Rabbit anti-FN1	Sigma	F3648
Goat anti-SOX10	R&D Systems	AF2864
Rabbit anti-IBA1	WAKO	019-19741
Rat anti-CD3	Abcam	ab11089
Chicken anti-GAP43	Novus Biologicals	NBP1-92714

Supplemental Table 1. Antibody information.

Gene Symbol	Primer Sequence (5'-3')
Col1a1	F: ATGGATTCCAGTTCGAGTAGGC<br>R: CATCGACAGTGACGCTGTAGG
Col1a2	F: ATGCCTAGCAACATGCCAATC<br>R: CAGCAAAGTTCCCACCGAGA
Col3a1	F: TTTTGCAGTGATATGTGATGTT<br>R: GGATGGTGGTTTTCAGTTTA
Col4a1	F: GGACTACCTGGAACAAAAGGG<br>R: GCCAAGTATCTCACCTGGATCA
Col6a1	F: ACAGTGACGAGGTGGAGATCA<br>R: GATAGCGCAGTCGGTGTAGG
Fn1	F: CCAGTCCTACAACCAGTATTCTC<br>R: CTTCTCTGTCAGCCTGTACATC
Lama2	F: TGCTGTCCTGAATCTTGCTTC<br>R: AGCATTTGTAATCGGGTGTCTC
Lamb1	F: AGGAACCCGAGTTCAGCTAC<br>R: CACGTCGAGGTCACCGAAA
Lamc1	F: GGACTCCGCCCGAGGAATA<br>R: ACTTGAGACGCACATAGGTGA
Nid1	F: CGGGGATGACTTCGTCTCTC<br>R: GTGGTGACGTAGACTGCGT
Hspg2	F: CCAAATGCGCTGGACACATTC<br>R: CGGACACCTCTCGGAACTCT
18s	F: ACCGCAGCTAGGAATAATGGA<br>R: GCCTCAGTTCCGAAAACCA

Supplemental Table 2. qPCR primer sequences.
